# Trainee psychotherapists’ emotion recognition accuracy improves after training: emotion recognition training as a tool for psychotherapy education

**DOI:** 10.3389/fpsyg.2023.1188634

**Published:** 2023-07-20

**Authors:** Lillian Döllinger, Lennart Björn Högman, Petri Laukka, Tanja Bänziger, Irena Makower, Håkan Fischer, Stephan Hau

**Affiliations:** ^1^Department of Psychology, Stockholm University, Stockholm, Sweden; ^2^Department of Psychology and Social Work, Mid Sweden University, Östersund, Sweden; ^3^Evidens University College, Göteborg, Västergötland, Sweden

**Keywords:** emotion recognition accuracy, trainee psychotherapists, emotion in psychotherapy, multimodal emotion recognition, micro expression recognition, training emotion recognition, psychotherapy education

## Abstract

**Introduction:**

Psychotherapists’ emotional and empathic competencies have a positive influence on psychotherapy outcome and alliance. However, it is doubtful whether psychotherapy education in itself leads to improvements in trainee psychotherapists’ emotion recognition accuracy (ERA), which is an essential part of these competencies.

**Methods:**

In a randomized, controlled, double-blind study (*N* = 68), we trained trainee psychotherapists (57% psychodynamic therapy and 43% cognitive behavioral therapy) to detect non-verbal emotional expressions in others using standardized computerized trainings – one for multimodal emotion recognition accuracy and one for micro expression recognition accuracy – and compared their results to an active control group one week after the training (*n* = 60) and at the one-year follow up (*n* = 55). The participants trained once weekly during a three-week period. As outcome measures, we used a multimodal emotion recognition accuracy task, a micro expression recognition accuracy task and an emotion recognition accuracy task for verbal and non-verbal (combined) emotional expressions in medical settings.

**Results:**

The results of mixed multilevel analyses suggest that the multimodal emotion recognition accuracy training led to significantly steeper increases than the other two conditions from pretest to the posttest one week after the last training session. When comparing the pretest to follow-up differences in slopes, the superiority of the multimodal training group was still detectable in the unimodal audio modality and the unimodal video modality (in comparison to the control training group), but not when considering the multimodal audio-video modality or the total score of the multimodal emotion recognition accuracy measure. The micro expression training group showed a significantly steeper change trajectory from pretest to posttest compared to the control training group, but not compared to the multimodal training group. However, the effect vanished again until the one-year follow-up. There were no differences in change trajectories for the outcome measure about emotion recognition accuracy in medical settings.

**Discussion:**

We conclude that trainee psychotherapists’ emotion recognition accuracy can be effectively trained, especially multimodal emotion recognition accuracy, and suggest that the changes in unimodal emotion recognition accuracy (audio-only and video-only) are long-lasting. Implications of these findings for the psychotherapy education are discussed.

## 1. Introduction

Working with patients’ emotions is an essential part of almost all forms of psychotherapy (see, e.g., Greenberg and Safran, 1989; Ehrenreich et al., 2007; Hutchison and Gerstein, 2012; Hofman, 2015; Greenberg et al., 2019). At the same time, it can be very difficult for psychotherapists, particularly trainee psychotherapists, to help patients to identify, reflect upon and experience their emotions. This can have multiple reasons, some related to the patient’s individual abilities and characteristics and some related to the psychotherapist’s (e.g., mentalizing and reflective functioning, alexithymia or other perceptive difficulties, emotional and empathic competencies, intrapsychic or interpersonal biases). Psychotherapeutic encounters are complex and the therapeutic interplay is characterized by both verbal and non-verbal communication dimensions that mutually influence each other and the therapeutic exchange (see, e.g., Westland, 2015; Del Giacco et al., 2020). In the present study, we want to shed light on non-verbal aspects of emotion communication and perception, more specifically, on trainee psychotherapists’ ability to recognize non-verbal emotional expressions in others and how this ability can be trained as part of the psychotherapy education.

Beyond the explicit, verbal exchange about emotions, a psychotherapist’s ability to recognize and work with patients’ non-verbal emotional expressions is considered a very important asset in psychotherapy (see, e.g., Greenberg and Safran, 1989; Hutchison and Gerstein, 2012; Donovan et al., 2017). Non-verbal expressions of emotion can be displayed via various channels or modalities, for example, via facial expressions, bodily postures, or tone of voice (Bhatara et al., 2014; de Gelder et al., 2015; Wickham, 2016), and sometimes they are displayed only very briefly before being masked or modulated by the sender (so called micro expressions, see Ekman and Friesen, 1969; Ekman, 2003; Matsumoto and Hwang, 2018), which can make the correct identification or interpretation of emotional expressions difficult. The psychotherapist’s ability to read and correctly recognize patients’ non-verbal expressions could be beneficial for empathically understanding patients, psychological assessment, planning interventions and establishing a good therapeutic relationship. The present study is focusing on non-verbal emotion recognition accuracy (ERA) in multiple modalities (audio, video, audio-video) and facial micro expression ERA.

There is meta-analytic research linking psychotherapists’ empathic abilities to psychotherapy outcome and alliance (e.g., Elliott et al., 2011, 2018; Nienhuis et al., 2018) and it seems that empathy, like other therapist factors, is particularly relevant for predicting the therapy outcome of less experienced therapists (Elliott et al., 2011). Most of the empathy research does not use standardized objective measures of this concept, but self-reports or observer ratings. Assessing psychotherapists’ ERA, as the perceptive aspect of empathy, could help filling this research gap. In a, to our knowledge, first systematic study about psychotherapists’ non-verbal ERA and therapy results, Abargil and Tishby (2021) found that psychotherapists’ ERA moderates several therapy process and outcome variables, like target complaint improvement, client working alliance, client overall emotion regulation, and avoidant attachment to therapist, among others. Psychotherapists with higher ERA produced better results. To summarize, there is good scientific support for the positive influence of psychotherapists’ empathy on psychotherapy process and outcome, and first evidence to suggest a similar influence of psychotherapists’ ERA.

Research about how well psychotherapists actually recognize non-verbal emotional expressions in others is somewhat mixed. Some research does not find differences in ERA between counseling trainees and undergraduate students (Hutchison and Gerstein, 2012), or between psychotherapists and matched controls (Hassenstab et al., 2007). On the other hand, Pauza et al. (2010) compared psychotherapy trainees to coaching trainees, a normal population sample and patients with anxiety disorders and found that the psychotherapy trainees had higher ERA than the other groups. Whether psychotherapists, or trainee psychotherapists, are better at recognizing non-verbal emotional expressions in others or not, it seems clear that ERA is a good ability to possess as psychotherapist. Thus, clinical psychology and psychotherapy education programs would do good to try to support their trainees in gaining (even better) ERA. Machado et al. (1999) investigated experienced psychotherapists ERA in comparison to a control group of undergraduate psychology students who wanted to become psychotherapists and found that experienced therapists possessed superior ERA. This finding suggests that clinical experience and education might lead to increased ERA. However, in a study investigating ERA in the beginning and at the end of one and a half years of practical psychotherapy training (Döllinger et al., *submitted*), we did not find trainee psychotherapists to improve their multimodal ERA or micro expression ERA significantly more than an undergraduate control group. This finding implies that psychotherapy education *per se* does not lead to ERA improvements and suggests that ERA might need to be trained more explicitly to lead to significant and lasting improvements. This view is shared by other researchers studying emotional competencies (e.g., emotion recognition, empathy, emotional intelligence or interpersonal sensitivity) in clinical professionals (see, e.g., Blanch-Hartigan, 2011; Hall, 2011; Kaplowitz et al., 2011; Blanch-Hartigan and Ruben, 2013; Hall et al., 2014;^45,1^; Curtis, 2021).

Although there are studies that confirm that the ability to recognize non-verbal emotional expressions in others can be trained with the help of standardized training procedures (for overviews, see, e.g., Schlegel et al., 2017; Rebeschini et al., 2019; Döllinger et al., 2021), research on trainee psychotherapists’ ERA is sparse. This is surprising since education about ERA and training of this ability have the potential to support trainee psychotherapists in working with patients’ emotions and to help secure optimal and safe treatments for patients (see, e.g., Heesacker and Bradley, 1997, for a discussion about possible reasons for that).

To our knowledge, there are so far only two randomized controlled studies examining ERA training for psychotherapists. Curtis (2021), in a sample including both graduate level counseling students and undergraduate psychology students, showed that a computerized training for micro expression detection improved ERA from pretest to immediate (same day) posttest, compared to the control condition of only watching a therapy session video. More specifically, significant improvements happened for the detection of contempt, anger and fear, with no differences between the graduate counseling and the undergraduate psychology students. Johnsen (2018) reported an improvement in detecting a patient’s emotional expressions in a filmed therapy session for psychotherapists undergoing Ekman’s Subtle Expression Training Tool (Paul Ekman Group, 2022) at the two-weeks posttest, compared to those who did not receive any training. There are also some studies about ERA training for other health care professionals, mostly medical students and medical residents. Most of them found that (micro expression) ERA could be improved with the help of interventions (Riess et al., 2012; Ragsdale et al., 2016; Wickham, 2016; Yu et al., 2016). One study found improved micro expression ERA only for medical students with high communicative skills (Endres and Laidlaw, 2009). Another training for verbal and non-verbal ERA targeting health care providers was evaluated in a group of undergraduate students (Blanch-Hartigan, 2011) and was found to be effective. Other studies (Robbins et al., 1979; Riess et al., 2011) found no improvements; however, the interventions did not explicitly include ERA, but related skills, e.g., empathy, and relational and interpersonal skills (for a review, see also Blanch-Hartigan and Ruben, 2013).

To summarize, studies about ERA training rarely target psychotherapists, but there are some studies evaluating trainings for other health care professions. Many of these studies lack control groups or standardized ERA outcome measures, and some use trainings for related traits (like empathy) to improve ERA. The sample sizes were mostly small and trainings varied in their quality and length or ERA facet targeted (i.e., micro expressions or macro expressions). Most studies, especially the higher-quality studies that include control groups and the two studies that target psychotherapists (Johnsen, 2018; Curtis, 2021), find ERA improvements due to explicit training.

### 1.1. Present study

Psychotherapists’ ERA is important for psychotherapy process and outcome. However, it is somewhat doubtful that standard psychotherapy training programs lead to improvements in trainee psychotherapists’ ERA without explicitly training this ability. Standardized computerized ERA trainings have been shown to be effective tools for improving non-verbal emotion recognition skills, but there are only a few studies about training psychotherapists. Training ERA more systematically as part of psychotherapy programs could potentially be a useful and cost-efficient way for improving (trainee) psychotherapists’ ERA. The aim of the present study was to investigate whether standardized computerized ERA training would lead to improvements in ERA for trainee psychotherapists, 1 week after the last training session and in a long-term follow-up 1 year later. In this study, we investigated two related, but distinct facets of ERA: dynamic multimodal (audio, video, audio-video) ERA and facial micro expression ERA. Investigating and training multimodal ERA in psychotherapy contexts is relevant because, so far, research is focusing heavily on still pictures of facial (micro) expressions even though it is likely more ecologically valid to consider ERA as a dynamic and multifaceted process. Further, targeting single modalities (e.g., non-verbal auditory expressions) in individualized trainings might benefit psychotherapists that are having difficulties in certain modalities or that are working in settings that make stronger use of one modality over others, like prosody in classical psychoanalysis in a lying-down setting or in telehealth. Investigating and training micro expression ERA in psychotherapy contexts is relevant because those very brief (< 200 ms) expressions could provide the clinician with information about patients’ conflicting, hidden, repressed or dissociated emotions (see, e.g., Donovan et al., 2017) and about patients’ psychological status for risk assessment (e.g., for masked suicidal or aggressive intentions; see Ekman and Friesen, 1969). Further, emotional response patterns in the face can be evoked without conscious awareness (e.g., by watching positive or negative micro expressions, see Dimberg et al., 2000) and are contributing to various kinds of nonverbal interactions, likely also in psychotherapy. There are also associations between micro expressions and certain interventions in psychotherapy, as well as with the strength of the therapeutic alliance (see Datz et al., 2019).

The present study is a randomized, controlled, double-blind study aiming to replicate the findings of a previous, randomized controlled study (Döllinger et al., 2021) in which we found standardized computerized multimodal ERA training and standardized computerized micro expression ERA training to be effective in improving ERA at the one-week posttest in a sample of undergraduate students. Both trainings were compared to each other and to an active control training. The participants trained once weekly during a three-week period. The multimodal training improved multimodal ERA and the micro expression training improved micro expression ERA, but we did not find transfer effects between these two ERA facets. Low baseline ERA was associated with higher ERA improvements. In the present study, we applied the same trainings to a sample of trainee psychotherapists (one multimodal training group and one micro expression training group) and compared them to a group of trainee psychotherapists undergoing an active control training. The trainee psychotherapists trained in either psychodynamic psychotherapy (PDT) or cognitive behavioral therapy (CBT).

### 1.2. Hypotheses and exploration

We hypothesized that each ERA training would lead to stronger improvements in ERA 1 week after the last training session (posttest) compared to the other two trainings. More specifically, the trainee psychotherapists that trained in multimodal ERA would improve their multimodal ERA more than the micro expression training group and the active control group, and that the trainee psychotherapists that trained in micro expression ERA would improve their micro expression ERA more than the multimodal training group and the active control group.

We hypothesized that these improvements would be long-lasting, meaning that the training groups would remain superior in their respective ERA facet (multimodal or micro expression ERA) even 1 year later (follow-up).

In exploratory analyses, we also investigated a third, unrelated ERA facet as outcome: a task that investigates ERA in medical situations and incorporates non-verbal and verbal audio-visual stimuli simultaneously (*Patient Emotion Cue Test*; Blanch-Hartigan, 2011). Since we know from previous research that ERA baseline is associated with magnitude of improvement (Döllinger et al., 2021), we explored whether the ERA changes were predicted by ERA baseline scores and whether individuals with low ERA at pretest would profit more from the ERA trainings than individuals with high baseline ERA. Further, previous research shows that age and gender can influence ERA (see, e.g., Thompson and Voyer, 2014; Cortes et al., 2021). Therefore, we also explored the influence of age and gender on ERA. Some research suggests that affective state can lead to bias in the perception of emotional expressions (e.g., emotion congruent or emotion incongruent emotional expressions are recognized more accurately), even if the results are somewhat contradictory (see, e.g., Schmid and Mast, 2010; Manierka et al., 2021). Thus, we also explored the influence of affective state on ERA. Finally, even if we did not divide the trainee psychotherapists into PDT and CBT students for our main analyses, we explored ERA differences between those groups.

## 2. Materials and methods

### 2.1. Data collection

Data collection took place at three different time points throughout the psychotherapy education for clinical psychology students at Stockholm University. After an education in different psychological fields and schools, the students can choose whether they want to train in PDT or CBT. The practical psychotherapy education of the ten-term-long clinical psychologist program starts in term 7 and lasts until the end of term 9 (about 1.5 years). It consists of theoretical courses and practical work under supervision at the university clinic (for more information about the psychotherapy education, see Döllinger et al., submitted).

In the present study, the pretest occurred in the beginning of term 7 before or right in the beginning of clinical work (the CBT students started their clinical work about 2 months later than the PDT students). About 43 days after the pretest (*M* = 43.35, *SD* = 12.78, range = 7−78), the training phase started. The participants were instructed to train once per week (with a 7-day interval) during three consecutive weeks on a computer placed at the psychotherapy clinic. They performed the three training sessions without supervision and according to their personal schedule, however, they were reminded to train and to adhere to their schedule throughout the process. The average time interval between training sessions was 6.53 days (SD = 1.98, range = 0–15 days), thus, most, but not all, adhered to the schedule. The posttest occurred about 1 week after the last training session (*M* = 7.87 days, SD = 4.01, range = 3−27), according to the participants’ individualized schedules. Both the training and the posttest also occurred during term 7. Then, there was a follow-up measurement near the end of the psychotherapy education (term 9). The follow-up occurred about 1 year after the posttest (*M* = 11.89 months, *SD* = 0.31, range = 11.15–12.85). The study was approved by the *Swedish Ethical Review Authority* (dnr 2015/1948–31) and all participants provided written informed consent prior to participation. The study was preregistered at *Open Science Framework*.^1^

### 2.2. Participants

Initially, 68 healthy participants enrolled in the study and completed the pretest. However, eight dropped out before the training phase began due to personal reasons. Five more were lost before the follow-up measurement. All participants attended Stockholm University’s clinical psychology training program and studied either PDT or CBT (see *Data collection*). Recruitment included email lists and oral presentations of the project in psychotherapy courses. The participants were reimbursed with sandwiches, gift vouchers and course credits. The sample size was not specified in advance, instead we tried to include as many participants as possible out of three cohorts of students starting their practical education during three consecutive terms. After the pretest, the participants were randomized to either the multimodal ERA training, the micro expression ERA training or the active control training. Since gender can play a role in ERA (see, e.g., Thompson and Voyer, 2014; Hall et al., 2016), we stratified for gender. To have an even distribution of CBT and PDT students in the groups, we also stratified for psychotherapy approach. There were no significant age differences between the groups. See Table 1 for sample characteristics and analyses of group differences during the different timepoints.

**Table 1:** Sample characteristics: descriptive statistics (means, standard deviations, range, count, 95% confidence intervals) and group comparisons (one-way Kruskal Wallis ANOVA of ranks).

Measures	Multimodal training	Micro expression training	Control training	Total	*Statistic*	*Effect size*
	*M* (*SD*) range	*M* (*SD*) range	*M* (*SD*) range	*M* (*SD*) range	χ^2^	ε^2^ [95% CI]^b^
*Age*						
Pre	31 (7.06)	28.39 (4.92)	30.91 (6.29)	30.9 (6.18)	χ^2^(2) = 1.65 (*p* = 0.44)	ε^2^ = 0.02 [0.00, 0.17]
	22−44	22−41	24−44	22−44		
	(*n* = 23)	(*n* = 23)	(*n* = 22)	(n = 68)		
Post	30.83 (7.11)	28.57 (5.11)	31 (6.43)	30.10 (6.23)	χ^2^(2) = 1.12 *(p* = 0.57)	ε^2^ = 0.02 [0.00, 0.17]
	22−44	22−41	24−44	22−44		
	(*n* = 18)	(*n* = 21)	(*n* = 21)	(*n* = 60)		
Follow-up	30.31 (7.32)	28.45 (5.22)	30.89 (6.23)	29.84 (6.21)	χ^2^(2) = 1.07 *(p* = 0.59)	ε^2^ = 0.02 [0.00, 0.18]
	22−44	22−41	24−44	22−44		
	(*n* = 16)	(*n* = 20)	(*n* = 19)	(*n* = 55)		
	Count	Count	Count	Count	χ^2^	ε^2^
						[95% CI]^b^
*Gender*						
Pre	13 women, 10 men	14 women, 9 men	13 women, 9 men	40 women, 28 men	χ^2^(2) = 0.09 (*p* = 0.96)	ε^2^ = 0.00 [0.00, 0.11]
Post	9 women, 9 men	13 women, 8 men	12 women, 9 men	34 women, 26 men	χ^2^(2) = 0.55 *(p* = 0.76)	ε^2^ = 0.01 [0.00, 0.16]
Follow-up	8 women, 8 men	12 women, 8 men	10 women, 9 men	30 women, 25 men	χ^2^(2) = 0.39 *(p* = 0.82)	ε^2^ = 0.01 [0.00, 0.17]
*Therapy approach*						
Pre	PDT = 13, CBT = 10	PDT = 13, CBT = 10	PDT = 13, CBT = 9	PDT = 39, CBT = 29	χ^2^(2) = 0.04 *(p* = 0.98)	ε^2^ = 0.00 [0.00, 0.11]
Post	PDT = 11, CBT = 7	PDT = 13, CBT = 8	PDT = 12, CBT = 9	PDT = 36, CBT = 24	χ^2^(2) = 0.11 *(p* = 0.95)	ε^2^ = 0.00 [0.00, 0.12]
Follow-up	PDT = 9, CBT = 7	PDT = 13, CBT = 7	PDT = 11, CBT = 8	PDT = 33, CBT = 22	χ^2^(2) = 0.33 *(p* = 0.85)	ε^2^ = 0.01 [0.00, 0.15]

[95% CI]^b^: 95% Confidence Interval for ε^2^ effect size is based on 1,000 bootstrap resamples of the mean difference (percentile interval). Common standardized effect size estimates: ε^2^ = 0.01 (small), ε^2^ = 0.08 (moderate), ε^2^ = 0.26 (large). *p < 0.05, **p < 0.01, ***p < 0.001.

### 2.3. Materials and procedures

The present study investigates two separate but related ERA facets: Emotion recognition accuracy for emotions in multiple modalities and ERA for micro expressions of the face. For this reason, there were two main ERA outcome measures, one measure for multimodal ERA that is the primary outcome measure for the multimodal ERA training, and one measure for micro expression ERA that is the primary outcome measure for the micro expression ERA training. We also used a third, independent outcome measure that is assessing ERA in medical clinical situations and that is incorporating both verbal and non-verbal emotional expressions (see *Outcome measures*). Further, for exploratory reasons, we administered questionnaires about affective state (see *Other measures*) and other trait and state questionnaires that will be reported elsewhere.

### 2.4. Outcome measures

As multimodal ERA outcome measure, we used the Swedish version of the *Emotion Recognition Assessment in Multiple Modalities* test (ERAM; Laukka et al., 2021). The ERAM is the primary outcome measure for the multimodal ERA training. The ERAM is a computerized task that consists of 72 dynamic items that are divided into audio-only, video-only, and a combination of audio-video clips of actors displaying 12 emotional expressions. The clips were taken from the *Geneva Multimodal Emotion Portrayals* (GEMEP; Bänziger et al., 2012) and the emotions used were *(hot) anger*, *anxiety*, *despair*, *disgust*, *(panic) fear*, *interest*, *joy*, *pleasure*, *pride*, *relief*, *irritation*, and *sadness*. The participants’ task is to watch or listen to the clips (depending on the modality) and to judge which emotion was displayed by choosing from a predefined list of response alternatives (same as the emotions above) as fast and as accurately as possible. Even though the task includes auditory information, it is a non-verbal task, as the audio and audio-video modalities make use of a pseudo-language (e.g., “ne kal i bam sud molen”), meaning that the prosody of the auditory information has to be associated with an emotion label and not the content (see Bänziger et al., 2012; Laukka et al., 2021). The ERAM provides the possibility to assess separate scores for the auditory (ERAM audio), visual (ERAM video) and audio-visual (ERAM audio-video) ERA modalities (à 24 items) as well as to calculate a combined score (ERAM). Further, the 12 emotions include basic as well as more subtle emotions and they vary regarding their valence and arousal levels. Thus, for descriptive reasons, we also calculated separate scores for valence and arousal according the GEMEP classification. Positive valence items were *interest*, *joy*, *pleasure*, *pride*, and *relief*; negative valence items were *anger, anxiety*, *despair*, *disgust*, *fear*, *irritation*, and *sadness*. High arousal emotions were *anger, fear, joy*, *pride,* and *despair*; low arousal emotions were *irritation*, *anxiety, pleasure*, *relief*, *interest*, and *sadness*. *Disgust* was not classified according to arousal. In this sample, the ERAM showed varying internal consistencies, from questionable to acceptable, depending on the time point (α_pre_ = 0.52; α_post_ = 0.71; α_follow up_ = 0.65). In two evaluation studies (Laukka et al., 2021), it showed better psychometric properties (α = 0.74; α = 0.80). In previous studies (Döllinger et al., submitted, Laukka et al., 2021), the ERAM also showed acceptable internal consistency and structure estimated with the omega coefficient (McDonald, 1999), but because of the lower sample size, omega was not computable for the present sample.

The measure for micro expression ERA was a computerized *micro expression recognition task* (MICRO; see Döllinger et al., 2021). The MICRO was the primary outcome measure for the micro expression ERA training. The micro expressions in this task were produced by showing a still picture of a facial emotional expression for 200 ms and double-masking it by a neutral facial expression (2 s). Each time, 70 emotion items out of a pool of 312 pictures from the *Radboud Faces Database* (Langner et al., 2010) were chosen randomly and presented to the participant that had to judge as fast and accurately as possible which emotion was briefly displayed. The pictures consisted of the faces of young female and male actors that were trained to produce emotional expressions according to the *Facial Action Coding System* (FACS; Ekman et al., 2002). The emotions in the MICRO were seven basic emotions (see Ekman and Cordaro, 2011): *happiness*, *surprise*, *fear*, *disgust*, *sadness*, *anger*, and *contempt*. Yet, due to a coding error, the micro expression training did not include the emotion *anger*. Because anger was not trained, we excluded those items also from the micro expression outcome measure MICRO. The MICRO was conducted in Swedish. The reliability of the MICRO (without anger items) was acceptable to good according to Cronbach’s alpha in the present study (α_pre_ = 0.71, α_post_ = 0.88, α_follow up_ = 0.87).

Further, a third, independent ERA outcome measure was used to assess ERA in clinical situations – a slightly modified version of the *Patient Emotion Cue Test* (PECT; Blanch-Hartigan, 2011). The PECT is a valid and reliable measure to assesses accuracy for recognizing combined verbal and non-verbal displays of emotion that are typical for the medical context. The participants watched 47 video clips (averaging 3 s each) in which a young female actor displayed one of five emotions (*anger*, *sadness*, *happiness*, *anxiety*, and *confusion*) or a neutral expression and was to indicate on a sheet of paper which emotion was shown, as well as rate the intensity of the verbal and non-verbal expression. The video clips included verbal statements that could take place in medical interactions (e.g., “It’s just being gradually getting worse”), thus, the PECT is a measure of verbal and non-verbal ERA. Internal consistencies for the PECT in the present study were acceptable for pre and posttest (α_pre_ = 0.70, α_post_ = 0.73), but not for the follow-up (α_follow up_ = 0.46).

### 2.5. Other measures

As measure of explicit affectivity, we used the *Positive and Negative Affect Schedule* (PANAS; Watson et al., 1988). In this sample, both the positive subscale (α_pos_pre_ = 0.82, α_pos_post_ = 0.84 and α_pos_follow up_ = 0.75) and the negative subscale of the PANAS (α_neg_pre_ = 0.75, α_neg_post_ = 0.86 and α_neg_follow up_ = 0.79) showed acceptable to good internal consistencies. Further, we used the *Implicit Positive and Negative Affect Test* (IPANAT; Quirin et al., 2009) as an indirect measure of positive and negative affectivity. In this task, the participants have to rate on a 4-point Likert scale (1 = *does not fit at all* – 4 = *fits very well*) how well artificial words (e.g., SAFME) convey positive and negative moods (*happy*, *energetic*, *cheerful*, *helpless*, *tense*, *inhibited*). Depending on how the individual participant judges the artificial words as positive or negative, state and trait affectivity is concluded. In this sample, the positive subscale of IPANAT showed questionable to good reliability depending on measurement time point (α_pos_pre_ = 0.81, α_pos_post_ = 0.84, α_pos_follow up_ = 0.66), and the negative subscale showed acceptable reliability (α_neg_pre_ = 0.77, α_neg_post_ = 0.79, α_neg_follow up_ = 0.72).

### 2.6. Lab procedures

The pretest, posttest and follow-up measurements at the lab started with self-reports about state affectivity (PANAS and IPANAT), followed by the three ERA tasks. The participants first completed the MICRO, then the ERAM and then the PECT. The test leaders in the lab that assisted the participants with the three ERA measurements were blind to the participants’ training conditions. After the follow-up measurement, the participants were asked whether they thought they belonged to the experimental group or the control group and were asked for comments about the training. The participants of the control group were debriefed about not having received an ERA training and given the opportunity to participate in one of the ERA trainings, however, none made use of this offer.

### 2.7. Trainings

The ERA trainings that were used in the present study have already been tested in a previous sample of undergraduate students and have been shown to be highly effective (Döllinger et al., 2021). For the training phase, the participants were instructed to train once per week during three consecutive weeks. The participants trained individually on a computer at facilities of the outpatient clinic and were blind to their condition. The duration of the ERA trainings was 10–15 min each. In the first training session, the multimodal training group and the micro expression training group started by watching a circa 10-min long video lecture about emotions and emotional expressions including theories of emotion and examples of facial expressions as well as about the relevance of ERA training for human interactions in different contexts. After that, the participants administered their respective ERA training independently and without supervision. However, they could always reach out to the test leader and were reminded to train and to follow the instructions on a regular basis. Each ERA training session took about 15 min; however, in the first session, the two ERA training groups also watched a video lecture about ERA, which prolonged the first session. The control training group did not watch the video lecture.

The *multimodal ERA training* consisted of training ERA separately for audio, video and audio-video stimuli. The items were taken from the extended GEMEP corpus (Bänziger et al., 2012) and the procedure was based on the ERAM (Laukka et al., 2021), though not using any items that were part of the ERAM. There were three training sessions and each session, 72 items were randomly chosen from a pool of 144 items (two items per emotional expression per modality). The emotions used were *anger*, *anxiety*, *despair*, *disgust*, *fear*, *interest*, *joy*, *pleasure*, *pride*, *relief*, *irritation*, and *sadness*. The task followed the same procedure as the ERAM. After watching and/or listening to an emotional expression, the participants had to indicate which emotion was displayed from a list of answering options. If the answer was correct, the participant received feedback on that. If not, then the participant was provided with information about the correct answer. After each training session, the participants also received extended feedback about which emotions they tended to mix up in form of a confusion matrix.

The *micro expression ERA training* specifically trained the ability to correctly recognize very brief (>200 ms) facial expressions and followed the procedure of the MICRO, but used a different item database, the *Karolinska Directed Emotional Faces* (Lundqvist et al., 1998). Each of the three training sessions consisted of 60 items that were randomly chosen from a pool of 336 items. After watching an image of an emotional expression that was double-masked with a neutral expression, the participants had to indicate which emotion was briefly displayed using a list of possible emotions (*happiness*, *surprise*, *fear*, *disgust*, *sadness*, *anger* or *contempt*). No images of angry expressions were included in the training due to a coding error. Thus, anger was not trained and was excluded from the analyses. After each choice, the participants received immediate feedback about whether their answer was correct and, in case of a wrong answer, what the correct answer would have been. In the end of each session, the participants received extended feedback in form of recognition rates per emotion.

As *active control training*, we used a working memory task with *N*-back format that gave immediate feedback during the learning phase (see Gerhardsson et al., 2019). The task consisted of deciding whether an emotionally laden picture from the *International Affective Picture System* (Lang et al., 2008) had been displayed before or not. Beyond the learning phase, there was no immediate or extended feedback. The task did not address emotion recognition in any way. However, it was used in the hope of being related to the topic closely enough to not be detected as the control condition. The task was comparable in duration to the ERA trainings (apart from the video lecture).

## 3. Data analysis

Data preparation and analyses were performed using *R* (R Core Team, 2022, v. 4.2.2) and *RStudio* (RStudio Team, 2022, v. 7.2). For the emotion recognition data, we used Wagner’s (1993) unbiased hitrate (H_u_) instead of the raw hitrate (average correct), which is a way of controlling for how often the individual participant used an emotion category incorrectly (controlling for response bias). The alpha level for significance tests was set at 5%, but for transparency we also report exact *p*-values. For analyzing differences between the three groups in regard to sample characteristics and ERA during the various test time points, we conducted parametric one-way Analyses of Variance (ANOVA) and nonparametric Kruskal-Wallis one-way ANOVA of ranks (Holm adjusted). Standardized effect size estimates (Eta squared and Epsilon squared) were interpreted according to the following common guidelines: η^2^ = 0.01 (small), η^2^ = 0.06 (moderate), η^2^ = 0.14 (large); and, ε^2^ = 0.01 (small), ε^2^ = 0.08 (moderate), ε^2^ = 0.26 (large). To test for possible influences of age, gender, affective state and psychotherapy approach on ERA, we performed simple and multiple linear regression analyses (see Supplementary Material) and Student’s *t*-tests. The internal consistencies of the ERA tasks were calculated using the KR-20 formula for dichotomous data (Kuder and Richardson, 1937) and Cronbach’s alpha was used for the reliability analyses of the questionnaire data. Omega (ω; McDonald, 1999) was not computable.

Because of the three measurement time points and dropout, we decided to analyze the data with mixed multilevel modeling. We applied a step-wise modeling procedure (see, e.g., Field and Wright, 2011; Finch et al., 2019) and compared the model fit based on Akaike’s information criterion (AIC), a goodness-of-fit estimate that corrects for model complexity. Analysis of variance was used to test for statistical differences between the models. To handle missing data, we used maximum likelihood estimation (Enders, 2011, 2022). We modeled an unconditional means model with a random intercept for the individual ERA scores, an unconditional growth model with random intercept and fixed slope, an unconditional growth model with random intercept and random slope (allowing the ERA trajectories to vary), and the conditional growth model with a random intercept, random slope and *training group* as time-invariant predictor. Time was anchored at baseline (pretest = 0, posttest = 1, follow-up = 2). Since the time interval between posttest and follow-up was much larger than the interval between pretest and posttest (1 week), and because we wanted to assess the ERA change differences between the three groups 1 week after the training and at the one-year follow-up as separate research questions, we used time as categorical variable. We did not specify a variance–covariance structure. In the ERAM analyses, the multimodal training group was used as center for the analysis (multimodal training = 1, micro expression training = 2, control training = 3) whereas for the MICRO analyses, we used the micro expression group as center (micro expression training = 1, multimodal training = 2, control training = 3). For the PECT analyses we used the control group as center (control training = 1, multimodal training = 2, micro expression training = 3). To answer *hypotheses 1* and *2*, we consulted the fixed effects of the *time* by *training group* interactions that provide information about the differences in ERA change trajectories. We used Feingold’s (2009, 2013) method (including the within differences and pooled standard deviations at pretest) to estimate standardized effect sizes for the group differences in ERA change trajectories (from pretest to posttest and from pretest to follow-up). The standardized effect sizes were interpreted according to Cohen’s (1988) suggestions: *d* = 0.2 (small), *d* = 0.5 (moderate) and *d* = 0.8 (large). Since the ERAM allows separate scores for the three modalities (audio, video, audio-video) and since the multimodal training consisted of unimodal and multimodal modalities, we also explored between-group differences in change trajectories for the three modalities separately. To investigate whether low baseline ERA was predicting a larger ERA improvement, we conducted logistic regression analyses for which we divided the participants of the training groups into high and low responders using median split.

Beyond *R*’s base packages, we used the following *R* packages: *apaTables* (Stanley, 2021), *car* (Fox and Weisberg, 2019), *DescTools* (Signorell et al., 2021), *dplyr* (Wickham et al., 2022), *effectsize* (Ben-Shachar et al., 2020), e*mmeans* (Lenth, 2023), *ggplot2* (Wickham, 2016), *ggpubr* (Kassambara, 2023), *nlme* (Pinheiro et al., 2022), *psych* (Revelle, 2022), *rcompanion* (Mangiafico, 2023), *reshape2* (Wickham, 2007), *rstatix* (Kassambara, 2021), *sjstats* (Lüdecke, 2021), *tidyr* (Wickham and Girlich, 2022), *validateR* (Desjardins, 2022).

## 4. Results

Table 2 displays descriptive statistics (observed scores) and group comparisons for the three ERA measures during the three measurement times. For the ERAM, we also report values for the three modalities and the valence and arousal categories. There were no ERA differences between the three groups at the pretest (*N* = 68). However, 1 week after the third training session, at posttest (*n* = 60), there were significant group differences in all ERAM variables and the MICRO, but not for the PECT. This suggests that the two trainings (multimodal ERA training and micro expression ERA training) lead to improvements in ERA and that the ERA facet that was not trained, verbal and non-verbal (combined) ERA in medical contexts (as assessed with the PECT) was not affected by any of the trainings. The only significant group difference at follow-up (*n* = 55) was in the audio modality of the ERAM, suggesting that the multimodal training group has retained superiority in this modality even 1 year after the training. It should be noted, though, that the 90% confidence interval of the effect includes 0. The results of the mixed multilevel models investigating the differences in ERA change trajectories (*time* by *group* interactions) are reported below.

**Table 2:** ERA test variables: descriptive statistics (means, standard deviations, 95% confidence intervals, sample size) and group comparisons (one-way ANOVA and one-way Kruskal Wallis ANOVA of ranks).

Measures	Multimodal training	Micro expression training	Control training	Total	Statistic	Effect size
	*M* (*SD*) [95% CI]	*M* (*SD*) [95% CI]	*M* (*SD*) [95% CI]	*M* (*SD*) [95% CI]	*F* / χ^2^	η^2^ / ε^2^ [90% CI] / [95% CI]^b^
**PRE (*N* = 68)**	***n* = 23**	***n* = 23**	***n* = 22**			
ERAM	0.46 (0.08) [0.43, 0.50]	0.46 (0.11) [0.41, 0.50]	0.45 (0.08) [0.41, 0.48]	0.46 (0.09) [0.43, 0.48]	*F*(2,65) = 0.23 (*p* = 0.79)	η^2^ = 0.01 [0.00, 0.05]
ERAM audio	0.42 (0.11) [0.37, 0.47]	0.42 (0.13) [0.37, 0.48]	0.42 (0.09) [0.38, 0.45]	0.42 (0.11) [0.39, 0.44]	*F*(2,66) = 0.03 (*p* = 0.87)	η^2^ = 0.00 [0.00, 0.00]
ERAM video	0.46 (0.12) [0.41, 0.51]	0.47 (0.09) [0.43, 51]	0.46 (0.14) [0.40, 52]	0.46 (0.12) [0.43, 0.49]	*F*(2,65) = 0.12 (*p* = 0.89)	η^2^ = 0.00 [0.00, 0.03]
ERAM audio-video	0.67 (0.10) [0.62, 0.71]	0.61 (0.16) [0.55, 0.68]	0.62 (0.13) [0.56, 0.68]	0.63 (0.13) [0.60, 0.66]	*F*(2,65) = 1.06 (*p* = 0.35)	η^2^ = 0.03 [0.00, 0.11]
ERAM positive valence	0.47 (0.13) [0.41, 0.53]	0.47 (0.12) [0.42, 0.53]	0.47 (0.12) [0.42, 0.52]	0.47 (0.12) [0.44, 0.50]	χ^2^(2) = 0.26 (*p* = 0.88)	ε^2^ = 0.00 [−0.03, 0.10]^b^
ERAM negative valence	0.46 (0.10) [0.41, 0.50]	0.45 (0.12) [0.39, 0.50]	0.43 (0.09) [0.39, 0.47]	0.44 (0.10) [0.42, 0.47]	*F*(2,65) = 0.48 (*p* = 0.62)	η^2^ = 0.01 [0.00, 0.07]
ERAM high arousal	0.47 (0.10) [0.42, 0.51]	0.47 (0.11) [0.42, 0.51]	0.42 (0.10) [0.38, 0.47]	0.45 (0.10) [0.43, 0.48]	*F*(2,65) = 1.28 (*p* = 0.29)	η^2^ = 0.04 [0.00, 0.12]
ERAM low arousal	0.64 (0.08) [0.61, 0.67]	0.65 (0.11) [0.60, 0.69]	0.64 (0.09) [0.60, 0.68]	0.64 (0.09) [0.62, 0.67]	*F*(2,65) = 0.05 (*p* = 0.96)	η^2^ = 0.00 [0.00, 0.00]
MICRO	0.51 (0.15) [0.44, 0.57]	0.57 (0.17) [0.49, 0.64]	0.51 (0.17) [0.44, 0.58]	0.53 (0.16) [0.49, 0.57]	*F*(2,65) = 0.94 (*p* = 0.40)	η^2^ = 0.03 [0.00, 0.10]
PECT	0.44 (0.09) [0.41, 0.48]	0.45 (0.11) [0.41, 0.50]	0.46 (0.11) [0.41, 0.51]	0.45 (0.10) [0.43, 0.48]	*F*(2,65) = 0.17 (*p* = 0.68)	η^2^ = 0.00 [0.00, 0.02]
**POST (*n* = 60)**	***n* = 18**	***n* = 21**	***n* = 21**			
ERAM	0.62 (0.06) [0.59, 0.65]	0.51 (0.11) [0.47, 0.57]	0.47 (0.11) [0.42, 0.52]	0.53 (0.11) [0.50, 0.56]	*F*(2,57) = 12.13 (*p* < 0.001***)	η^2^ = 0.30 [0.13, 0.44]
ERAM audio	0.60 (0.13) [0.54, 0.67]	0.48 (0.12) [0.43, 0.54]	0.42 (0.13) [0.36, 0.48]	0.50 (0.14) [0.46, 0.53]	*F*(2,57) = 10.92 (*p* < 0.001***)	η^2^ = 0.28 [0.11, 0.42]
ERAM video	0.62 (0.11) [0.57, 0.68]	0.54 (0.12) [0.48, 0.59]	0.49 (0.14) [0.43, 0.56]	0.55 (0.13) [0.51, 0.58]	*F*(2,57) = 5.45 (*p* = 0.01**)	η^2^ = 0.16 [0.03, 0.30]
ERAM audio-video	0.74 (0.09) [0.69, 0.79]	0.66 (0.15) [0.59, 0.73]	0.62 (0.14) [0.55, 0.68]	0.67 (0.14) [0.63, 0.70]	*F*(2,57) = 4.29 (*p* = 0.02*)	η^2^ = 0.13 [0.01, 0.26]
ERAM positive valence	0.63 (0.13) [0.56, 0.69]	0.55 (0.15) [0.49, 0.62]	0.49 (0.13) [0.44, 0.55]	0.55 (0.14) [0.52, 0.59]	*F*(2,57) = 4.72 (*p* = 0.01**)	η^2^ = 0.14 [0.02, 0.27]
ERAM negative valence	0.62 (0.07) [0.58, 0.65]	0.49 (0.12) [0.44, 0.55]	0.45 (0.12) [0.39, 0.50]	0.51 (0.13) [0.48, 0.54]	*F*(2,57) = 12.55 (*p* < 0.001***)	η^2^ = 0.31 [0.14, 0.44]
ERAM high arousal	0.63 (0.09) [0.58, 0.67]	0.51 (0.13) [0.45, 0.57]	0.46 (0.12) [0.41, 0.52]	0.53 (0.13) [0.50, 0.56]	*F*(2,57) = 10.25 (*p* < 0.001***)	η^2^ = 0.27 [0.10, 0.40]
ERAM low arousal	0.77 (0.06) [0.74, 0.80]	0.71 (0.10) [0.67, 0.76]	0.65 (0.11) [0.60, 0.70]	0.71 (0.10) [0.68, 0.73]	*F*(2,57) = 7.44 (*p* < 0.001***)	η^2^ = 0.21 [0.06, 0.35]
MICRO	0.68 (0.13) [0.62, 0.74]	0.80 (0.13) [0.74, 0.86]	0.63 (0.12) [0.58, 0.69]	0.71 (0.14) [0.67, 0.74]	χ^2^(2) = 13.44 (*p* = 0.001***)	ε^2^ = 0.23 [0.04, 0.39]^b^
PECT	0.47 (0.15) [0.40, 0.54]	0.50 (0.12) [0.44, 0.55]	0.49 (0.14) [0.43, 0.55]	0.49 (0.13) [0.45, 0.52]	*F*(2,56) = 0.19 (*p* = 0.83)	η^2^ = 0.01 [0.00, 0.05]
**FOLLOW-UP (*n* = 55)**	***n* = 16**	***n* = 20**	***n* = 19**			
ERAM	0.55 (0.11) [0.49, 0.61]	0.53 (0.11) [0.48, 0.58]	0.49 (0.08) [0.45, 0.53]	0.52 (0.10) [0.49, 0.55]	*F*(2,52) = 1.63 (*p* = 0.06)	η^2^ = 0.06 [0.00, 0.17]
ERAM audio	0.56 (0.17) [0.47, 0.65]	0.49 (0.12) [0.43, 0.54]	0.44 (0.11) [0.39, 0.50]	0.49 (0.14) [0.46, 0.53]	*F*(2,52) = 3.35 (*p* = 0.04*)	η^2^ = 0.11 [0.00, 0.25]
ERAM video	0.57 (0.14) [0.50, 0.65]	0.55 (0.12) [0.49, 0.60]	0.60 (0.11) [0.44, 0.55]	0.54 (0.12) [0.50, 0.57]	*F*(2,52) = 1.89 (*p* = 0.16)	η^2^ = 0.07 [0.00, 0.18]
ERAM audio-video	0.67 (0.10) [0.62, 0.73]	0.67 (0.15) [0.60, 0.74]	0.67 (0.13) [0.60, 0.73]	0.67 (0.13) [0.63, 0.70]	*F*(2,52) = 0.01 (*p* = 0.99)	η^2^ = 0.00 [0.00, 0.00]
ERAM positive valence	0.58 (0.15) [0.50, 0.66]	0.55 (0.13) [0.49, 0.61]	0.48 (0.12) [0.43, 0.54]	0.54 (0.13) [0.50, 0.57]	*F*(2,52) = 2.68 (*p* = 0.08)	η^2^ = 0.09 [0.00, 0.22]
ERAM negative valence	0.53 (0.11) [0.47, 0.59]	0.51 (0.15) [0.44, 0.58]	0.50 (0.09) [0.45, 0.54]	0.51 (0.12) [0.48, 0.54]	*F*(2,52) = 0.41 (*p* = 0.67)	η^2^ = 0.02 [0.00, 0.08]
ERAM high arousal	0.56 (0.13) [0.49, 0.63]	0.53 (0.12) [0.47, 0.58]	0.51 (0.10) [0.47, 0.56]	0.53 (0.11) [0.50, 0.56]	*F*(2,52) = 0.74 (*p* = 0.48)	η^2^ = 0.03 [0.00, 0.11]
ERAM low arousal	0.73 (0.09) [0.68, 0.77]	0.71 (0.10) [0.67, 0.76]	0.66 (0.09) [0.62, 0.70]	0.70 (0.09) [0.67, 0.72]	*F*(2,52) = 2.55 (*p* = 0.09)	η^2^ = 0.09 [0.00, 0.21]
MICRO	0.55 (0.14) [0.48, 0.63]	0.65 (0.14) [0.58, 0.72]	0.52 (0.21) [0.42, 0.63]	0.58 (0.17) [0.53, 0.62]	*F*(2,52) = 2.92 (*p* = 0.06)	η^2^ = 0.10 [0.00, 0.23]
PECT	0.51 (0.09) [0.47, 0.56]	0.48 (0.12) [0.43, 0.54]	0.50 (0.09) [0.46, 0.54]	0.50 (0.10) [0.47, 0.52]	*F*(2,52) = 0.47 (*p* = 0.63)	η^2^ = 0.02 [0.00, 0.09]

Table 3 presents the observed scores. The ERA scores (range 0–1) are presented as unbiased hitrates (H_u_). For the mixed multilevel modeling analyses, missing data were handled via maximum likelihood estimation. [90% CI]: 90% Confidence Interval for η^2^ effect size. [95% CI]^b^: 95% Confidence Interval for ε^2^ effect size is based on 1,000 bootstrap resamples of the mean difference (percentile interval). Common standardized effect size estimates: η^2^ = 0.01 (small), η^2^ = 0.06 (moderate) and η^2^ = 0.14 (large); ε^2^ = 0.01 (small), ε^2^ = 0.08 (moderate), ε^2^ = 0.26 (large).

*p < 0.05, **p < 0.01, ***p < 0.001.

We tested whether there were any group differences regarding affective state (Table 3) and found that the micro expression group reported significantly higher positive mood according to the PANAS than the other two groups at the posttest. We performed linear regression analyses to explore a possible influence of explicit (PANAS) and implicit (IPANAT) affective state on ERA (see Supplementary Table S1). We found that the negative affective state scale of the PANAS predicted multimodal ERA at pretest (*b* = −0.06, *p* = 0.04, *SE_b_* = 0.03, β = −0.26, *R*^2^ = 0.07). Since there were no group differences in multimodal ERA at pretest, we did not explore this further. Micro expression ERA at posttest was predicted by negative affective state as measured by the PANAS (*b* = −0.08, *p* = 0.04, *SE_b_* = 0.04, β = 0.27, *R*^2^ = −0.07), in so far that micro expression ERA increased with increasing negative mood, and by positive affective state as measured by the IPANAT (*b* = −0.13, *p* = 0.01, *SE_b_* = 0.05, β = −0.33, *R*^2^ = 0.11), in so far that micro expression ERA decreased, the more positive the participants felt. We followed up on this in an additional linear regression analysis for the MICRO post score (see the results for *Micro expression ERA* and Supplementary Table S3). Linear regression analyses exploring the influence of age and gender on ERA suggest that participants’ age predicted how they scored on the PECT at pretest (*b* = −0.00, *p* = 0.04, *SE_b_* = 0.00, β = −0.28, *R*^2^ = 0.08). The ERA for verbal and non-verbal (combined) emotional expressions in medical settings (according to the PECT) decreases with age (Supplementary Table S2). However, since this was the only significant age or gender prediction and since the PECT is not a primary outcome measure of this study, the finding should not be overstated. In the *Supplementary Material*, the reader finds exploratory analyses about ERA group differences between PDT and CBT trainees (Supplementary Table S5) and the individual ERA trajectories of all participants (observed data, Supplementary Figures S1–S3). The only significant influence of psychotherapy approach on ERA was for the PECT at follow-up, in which the CBT students performed significantly better.

**Table 3:** Affective state measures: descriptive statistics (means, standard deviations, range, count, 95% confidence intervals) and group comparisons (one-way ANOVA and one-way Kruskal Wallis ANOVA of ranks).

Measures	Multimodal training	Micro expression training	Control training	Total	Statistic	Effect size
	*M* (*SD*) [95% CI]	*M* (*SD*) [95% CI]	*M* (*SD*) [95% CI]	*M* (*SD*) [95% CI]	*F* / χ^2^	η^2^ / ε^2^ [90% CI] / [95% CI]^b^
*State Affect (PANAS)*						
Pre: Positive	3.18 (0.59)	3.20 (0.39)	3.10 (0.70)	3.16 (0.56)	*F* (2,61) = 0.21 (*p* = 0.82)	η^2^ = 0.01 [0.00, 0.04]
	[2.90, 3.46]	[3.03, 3.37]	[2.78, 3.41]	[3.02, 3.30]		
	(*n* = 20)	(*n* = 23)	(*n* = 21)	*(n* = 64)		
Pre: Negative	1.46 [0.39)	1.40 [0.47)	1.48 [0.30)	1.45 [0.39)	*χ*^2^[2) = 1.77 (*p* = 0.41)	ε^2^ = 0.03 [0.00, 0.02]
	[1.28, 1.64]	[1.20, 1.60]	[1.34, 1.62]	[1.35, 1.54]		
	(*n* = 20)	(*n* = 23)	*(n* = 21)	*(n* = 64)		
Post: Positive	2.60 (0.49)	3.04 (0.63)	2.75 (0.57)	2.81 (0.59)	*χ*^2^(2) = 7.25 (*p* = 0.03)*	ε^2^ = 0.03 [0.00, 0.09]
	[2.35, 2.85]	[2.74, 3.34]	[2.49, 3.01]	[2.65, 2.96]		
	(*n* = 17)	(*n* = 20)	(*n* = 21)	*(n* = 58)		
Post: Negative	1.42 (0.39)	1.49 (0.58)	1.33 (0.36)	1.41 (0.45)	*χ*^2^(2) = 0.66 (*p* = 0.72)	ε^2^ = 0.00 [0.00, 0.04]
	[1.22, 1.61]	[1.21, 1.76]	[1.17, 1.50]	[1.29, 1.53]		
	(*n* = 18)	(*n* = 20)	*(n* = 21)	*(n* = 59)		
Follow-up: Positive	2.46 (0.49)	2.71 (0.38)	2.72 (0.62)	2.64 (0.51)	*F* (2,61) = 1.43 (*p* = 0.25)	η^2^ = 0.05 [0.00, 0.15]
	[2.20, 2.72]	[2.54, 2.89]	[2.41, 3.03]	[.50, 2.78]		
	(*n* = 16)	(*n* = 20)	(*n* = 18)	(*n* = 54)		
Follow-up: Negative	1.38 (0.40)	1.52 (0.47)	1.34 (0.36)	1.42 (0.41)	*χ*^2^(2) = 1.93 (*p* = 0.38)	ε^2^ = 0.01 [0.00, 0.07]
	(1.17, 1.59]	(1.30, 1.74]	[1.16, 1.51]	[1.30, 1.53]		
	(*n* = 16)	(*n* = 20)	(*n* = 19)	(*n* = 55)		
*State Affect (IPANAT)*						
Pre: Positive	1.99 (0.37)	1.92 (0.31)	2.04 (0.45)	1.98 (0.37)	*F* (2,58) = 0.56 (*p* = 0.57)	η^2^ = 0.02 [0.00, 0.09]
	[1.82, 2.16]	[1.78, 2.06]	[1.83, 2.26]	[1.88, 2.07]		
	(*n* = 20)	(*n* = 22)	(*n* = 19)	*(n* = 61)		
Pre: Negative	1.98 (0.45)	1.89 (0.35)	1.96 (0.40)	1.94 (0.40)	*F* (2, 57) = 0.33 (*p* = 0.72)	η^2^ = 0.01 [0.00, 0.07]
	[1.76, 2.21]	[1.74, 2.04]	[1.77, 2.16]	[1.84, 2.04]		
	(*n* = 18)	(*n* = 23)	(*n* = 19)	*(n* = 60)		
Post: Positive	2.07 (0.37)	1.94 (0.27)	2.10 (0.40)	2.04 (0.35)	*F* (2, 53) = 1.07 (*p* = 0.35)	η^2^ = 0.04 [0.00, 0.13]
	[1.88, 2.25]	[1.80, 2.07]	[1.91, 2.28]	[1.94, 2.13]		
	(*n* = 18)	(*n* = 18)	(*n* = 20)	*(n* = 56)		
Post: Negative	1.94 (0.34)	1.94 (0.35)	1.73 (0.30)	1.87 (0.34)	*F* (2, 49) = 2.50 (*p* = 0.09)	η^2^ = 0.09 [0.00, 0.22]
	[1.76, 2.11]	[1.76, 2.13]	[1.58, 1.87]	[1.77, 1.96]		
	(*n* = 17)	(*n* = 17)	*(n* = 18)	*(n* = 52)		
Follow-up: Positive	2.05 (0.27)	2.00 (0.32)	2.11 (0.30)	2.05 (0.29)	*F* (2,48) = 0.59 (*p* = 0.56)	η^2^ = 0.02 [0.02, 0.11]
	[1.91, 2.20]	[1.85, 2.15]	[1.95, 2.27]	[1.97, 2.13]		
	(*n* = 15)	(*n* = 20)	(*n* = 16)	(*n* = 51)		
Follow-up: Negative	1.95 (0.43)	2.07 (0.28)	1.86 (0.30)	1.97 (0.34)	*F* (2,48) = 1.87 (*p* = 0.17)	η^2^ = 0.07 [0.07, 0.19]
	[1.71, 2.20]	[1.94, 2.20]	[1.70, 2.01]	[1.87, 2.06]		
	(*n* = 14)	(*n* = 20)	(*n* = 17)	(*n* = 51)		

Some data loss for the PANAS and IPANAT due to technical reasons and incomplete data. [90% CI]: 90% Confidence Interval for η^2^ effect size. [95% CI]^b^: 95% Confidence Interval for ε^2^ effect size is based on 1,000 bootstrap resamples of the mean difference (percentile interval). Common standardized effect size estimates: η^2^ = 0.01 (small), η^2^ = 0.06 (moderate), η^2^ = 0.14 (large); ε^2^ = 0.01 (small), ε^2^ = 0.08 (moderate), ε^2^ = 0.26 (large).*p < 0.05, **p < 0.01, ***p < 0.001.

The experimental check at the end of the follow-up measurement showed that most participants accurately perceived whether they had received an ERA training or a control training. Eighty-one percent of the participants of the control training group stated that they thought they were a part of the control group (19% thought they had received an actual ERA training); of the multimodal training group, 75% thought they had received an ERA training (19% thought they had received a control training, one participant did not answer); and of the micro expression training group, 65% thought they had received an ERA training (35% thought they had received a control training).

### 4.1. Multimodal ERA

In the mixed multilevel analysis for ERA in multiple modalities (ERAM), the conditional growth model with a random intercept, fixed slope and *training group* as time-invariant predictor had the best model fit (AIC = -382.19) compared to the unconditional means model with a random intercept (AIC = −327.68), the unconditional growth model with random intercept and fixed slope (AIC = −362.88), and the unconditional growth model with random intercept and random slope (AIC = −359.89). The conditional growth model was significantly different (*p* < 0.001) from the other models and the data under the conditional growth model was about 31 times more likely than under the next best model (unconditional growth model with random intercept and fixed slope) according to a Likelihood Ratio Test, χ^2^(6) = 31.32, *p* < 0.001).

**Figure 1:**
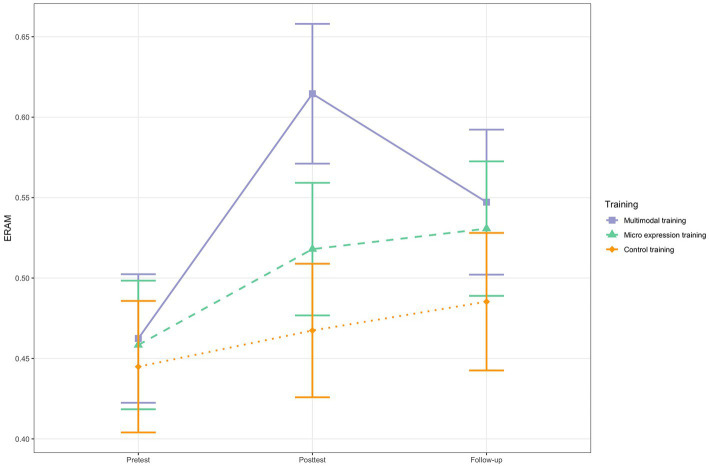
ERAM change for the three training groups. *N* = 68. Based on estimated marginal means. Error bars represent 95% Confidence Intervals.

At pretest, all three groups showed equivalent ERA (Table 2). Table 4 shows the fixed effects of the conditional growth model with *time* by *training group* interactions. There were significant interaction effects regarding the multimodal ERA changes from pretest to posttest. The multimodal training group showed significantly steeper multimodal ERA increases from pretest to posttest than the micro expression training group (between-group difference in slope = −0.09, *SE* = 0.03, *t*(109) = −3.36, *p* < 0.001; 95% CI [−0.15, −0.04]) and the control training group (between-group difference in slope = −0.13, *SE* = 0.03, *t*(109) = −4.69, *p* < 0.001; 95% CI [−0.18, −0.08]). See Figure 1 for a visual display. The pretest-posttest difference in change scores was large (*d* = 0.90) for the comparison with the micro expression training group and very large (*d* = 1.63) for the comparison with the control training group. The multimodal training group significantly increased with 15% (within-group change) and the micro expression training group significantly increased with 6%, but the control training did not (see Table 5).

**Table 4:** ERAM: Fixed effects of the conditional growth model fit by maximum likelihood estimation.

	Value	*SE*	*Df*	*t*-value	*p value*	95% CI
Intercept	0.46	0.02	109	23.10	0.00***	0.42, 0.50
Time						
pre-posttest	0.15	0.02	109	7.57	0.00***	0.11, 0.19
pre-follow-up	0.08	0.02	109	4.04	0.00***	0.04, 0.13
Training						
MMT vs. MET	−0.00	0.03	65	−0.14	0.89	−0.06, 0.05
MMT vs. CT	−0.02	0.03	65	−0.61	0.54	−0.07, 0.04
Interactions						
pre-posttest: MMT vs. MET	−0.09	0.03	109	−3.36	0.00***	−0.15, −0.04
pre-follow-up: MMT vs. MET	−0.01	0.03	109	−0.44	0.66	−0.07, 0.04
pre-posttest: MMT vs. CT	−0.13	0.03	109	−4.69	0.00***	−0.18, −0.08
pre-follow-up: MMT vs. CT	−0.04	0.03	109	−1.54	0.13	−0.10, 0.01

Number of Observations: 183; Number of Groups: 68. MMT, Multimodal training; MET, Micro expression training; CT, Control training.

*p < 0.05, **p < 0.01, ***p < 0.001.

**Table 5:** ERA tasks: Marginal contrasts analyses (within-group changes).

Time contrast	Training	*Difference*	95% CI	*SE*	*t(109)*	*p*-value
ERAM						
pre-posttest	MMT	−0.15	−0.20, −0.10	0.02	−7.57	0.001***
pre-posttest	MET	−0.06	−0.11, −0.01	0.02	−3.16	0.01**
pre-posttest	CT	−0.02	−0.07, 0.02	0.02	−1.19	0.48
pre-follow-up	MMT	−0.08	−0.14, −0.03	0.02	−4.04	0.001***
pre-follow-up	MET	−0.07	−0.12, −0.03	0.02	−3.77	0.001***
pre-follow-up	CT	−0.04	−0.09, 0.01	0.02	−2.06	0.48
MICRO						
pre-posttest	MET	−0.24	−0.34, −0.14	0.04	−5.94	0.001***
pre-posttest	MMT	−0.17	−0.28, −0.07	0.04	−4.08	0.001***
pre-posttest	CT	−0.13	−0.23, −0.03	0.04	−3.13	0.01**
pre-follow-up	MET	−0.09	−0.19, 0.01	0.04	−2.13	0.04*
pre-follow-up	MMT	−0.05	−0.15, 0.06	0.04	−1.07	0.29
pre-follow-up	CT	−0.02	−0.12, 0.08	0.04	−0.42	0.68
PECT						
pre-posttest	CT	−0.03	−0.09, 0.03	0.02	−1.37	0.50
pre-posttest	MMT	−0.04	−0.10, 0.02	0.03	−1.61	0.22
pre-posttest	MET	−0.04	−0.10, 0.01	0.02	−1.85	0.20
pre-follow-up	CT	−0.03	−0.09, 0.03	0.02	−1.40	0.50
pre-follow-up	MMT	−0.08	−0.15, −0.02	0.03	−3.11	0.01**
pre-follow-up	MET	−0.03	−0.09, 0.03	0.02	−1.13	0.53

MET, Micro expression training; MMT, Multimodal training; CT; Control training.

*p < 0.05, **p < 0.01, ***p < 0.001 (Holm adjusted).

For all that, there were no significant interactions when considering the pretest to follow-up change trajectories (Table 4), suggesting that no group showed superior long-term change. The multimodal training group (8%) and the micro expression training group (7%) increased significantly in multimodal ERA from pretest to follow-up, while the control group did not (Table 5 and Figure 1). But the group differences in change trajectories from pretest to posttest were not stable.

**Figure 2:**
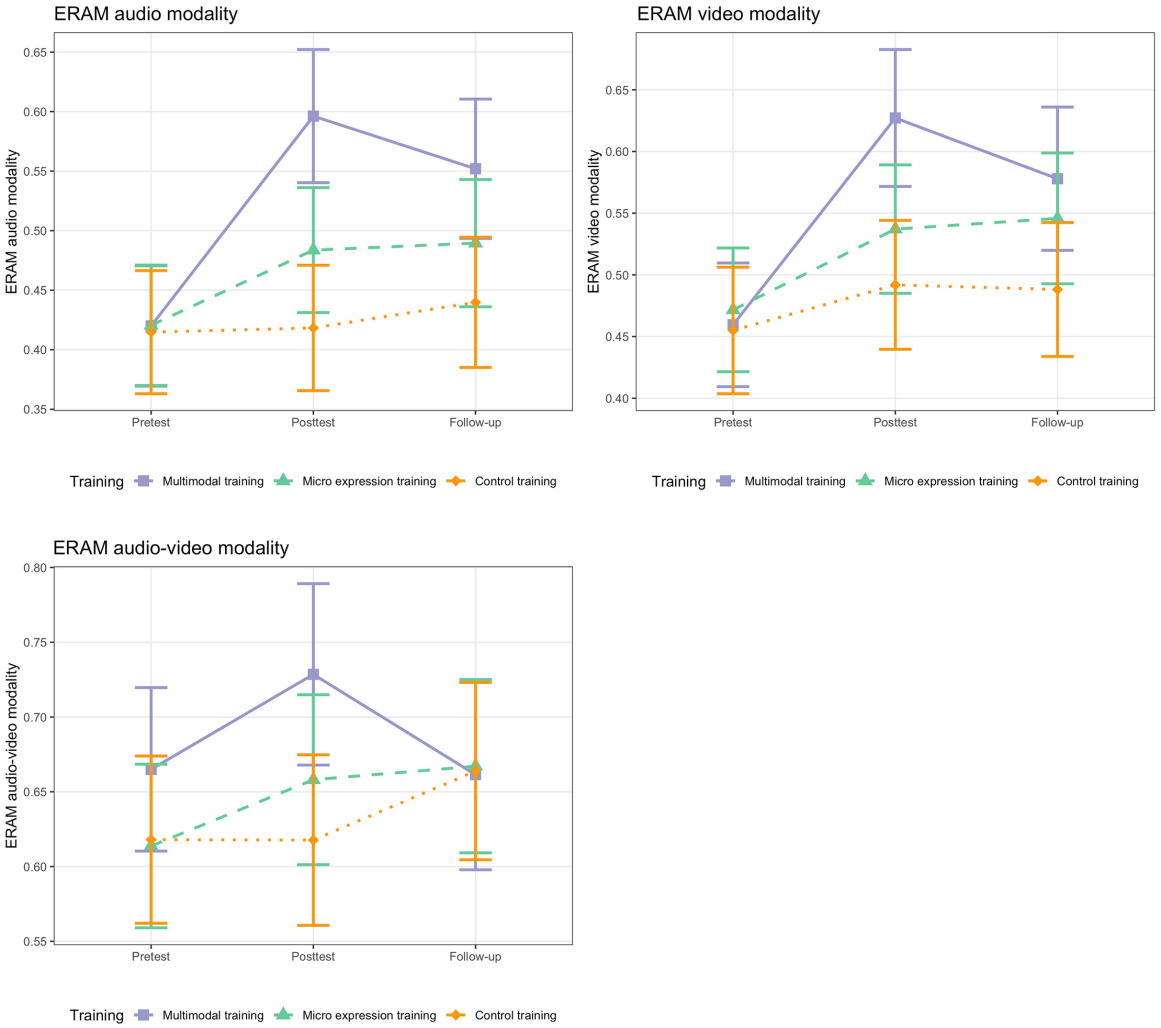
ERAM change for the three training groups per modality. *N* = 68. Based on estimated marginal means. Error bars represent 95% Confidence Intervals.

To explore whether there were different patterns for the three different ERAM modalities, we performed three exploratory mixed multilevel analyses using the modality scores as outcome (instead of the ERAM total score). The procedure was the same as for the ERAM (total score) analysis. Table 6 shows the fixed effects for the three ERAM modality models and Figure 2 provides a visual display of the change trajectories. See Table 7 for marginal contrast analyses.

**Table 6:** ERAM modalities: Fixed effects of the conditional growth model fit by maximum likelihood estimation.

	Value	*SE*	*Df*	*t*-value	*p value*	95% CI
**Audio-only modality**						
Intercept	0.42	0.03	109	16.59	0.00***	0.37, 0.47
Time						
pre-posttest	0.18	0.03	109	6.00	0.00***	0.12, 0.23
pre-follow-up	0.13	0.03	109	4.31	0.00***	0.07, 0.19
Training						
MMT vs. MET	0.00	0.04	65	0.02	0.99	−0.07, 0.07
MMT vs. CT	−0.01	0.04	65	−0.14	0.89	−0.08, 0.07
Interactions						
pre-posttest: MMT vs. MET	−0.11	0.04	109	−2.80	0.01**	−0.19, −0.04
pre-follow-up: MMT vs. MET	−0.06	0.04	109	−1.51	0.13	−0.14, 0.02
pre-posttest: MMT vs. CT	−0.17	0.04	109	−4.27	0.00***	−0.25, −0.09
pre-follow-up: MMT vs. CT	−0.11	0.04	109	−2.54	0.01**	−0.19, −0.03
**Video-only modality**						
Intercept	0.46	0.03	109	18.30	0.00***	0.41, 0.51
Time						
pre-posttest	0.17	0.03	109	5.74	0.00***	0.11, 0.22
pre-follow-up	0.12	0.03	109	3.89	0.00***	0.06, 0.18
Training						
MMT vs. MET	0.01	0.04	65	0.34	0.73	−0.06, 0.08
MMT vs. CT	−0.00	0.04	65	−0.13	0.90	−0.07, 0.07
Interactions						
pre-posttest: MMT vs. MET	−0.10	0.04	109	−2.55	0.01**	−0.18, −0.02
pre-follow-up: MMT vs. MET	−0.04	0.04	109	−1.07	0.29	−0.12, 0.04
pre-posttest: MMT vs. CT	−0.13	0.04	109	−3.25	0.00***	−0.21, −0.05
pre-follow-up: MMT vs. CT	−0.09	0.04	109	−2.04	0.04*	−0.17, −0.00
**Audio-video modality**						
Intercept	0.67	0.03	109	24.28	0.00***	0.61, 0.72
Time						
pre-posttest	0.06	0.03	109	1.95	0.05*	0.00, 0.13
pre-follow-up	−0.00	0.03	109	−0.10	0.92	−0.07, 0.06
Training						
MMT vs. MET	−0.05	0.04	65	−1.32	0.19	−0.13, 0.02
MMT vs. CT	−0.05	0.04	65	−1.20	0.23	−0.12, 0.03
Interactions						
pre-posttest: MMT vs. MET	−0.02	0.04	109	−0.43	0.67	−0.11, 0.07
pre-follow-up: MMT vs. MET	0.06	0.05	109	1.23	0.22	−0.03, 0.15
pre-posttest: MMT vs. CT	−0.06	0.04	109	−1.42	0.16	−0.15, 0.02
pre-follow-up: MMT vs. CT	0.05	0.05	109	1.06	0.29	−0.04, 0.14

Number of Observations: 183; Number of Groups: 68. MMT, Multimodal training; MET, Micro expression training; CT, Control training.

*p < 0.05, **p < 0.01, ***p < 0.001.

**Table 7:** ERAM modalities: Marginal contrasts analyses (within-group changes).

Time contrast	Training	*Difference*	95% CI	*SE*	*t (109)*	*p value*
Audio-only						
pre-posttest	MMT	−0.18	−0.25, −0.10	0.03	−6.00	0.001***
pre-posttest	MET	−0.06	−0.13, 0.00	0.03	−2.28	0.05*
pre-posttest	CT	−0.00	−0.07, 0.06	0.03	−0.13	0.99
pre-follow-up	MMT	−0.13	−0.21, −0.06	0.03	−4.31	0.001***
pre-follow-up	MET	−0.07	−0.14, 0.00	0.03	−2.45	0.05*
pre-follow-up	CT	−0.03	−0.10, 0.05	0.03	−0.87	0.99
Video-only						
pre-posttest	MMT	−0.17	−0.24, −0.10	0.03	−5.74	0.001***
pre-posttest	MET	−0.07	−0.13, 0.00	0.03	−2.37	0.04*
pre-posttest	CT	−0.04	−0.10, 0.03	0.03	−1.33	0.56
pre-follow-up	MMT	−0.12	−0.19, −0.04	0.03	−3.89	0.001***
pre-follow-up	MET	−0.07	−0.14, −0.01	0.03	−2.65	0.03*
pre-follow-up	CT	−0.03	−0.10, 0.04	0.03	−1.16	0.56
Audio-video						
pre-posttest	MMT	−0.06	−0.14, 0.02	0.03	−1.95	0.16
pre-posttest	MET	−0.04	−0.12, 0.03	0.03	−1.44	0.31
pre-posttest	CT	−0.00	−0.08, 0.08	0.03	−0.00	0.99
pre-follow-up	MMT	−0.00	−0.08, 0.09	0.03	−0.10	0.92
pre-follow-up	MET	−0.05	−0.13, 0.02	0.03	−1.71	0.27
pre-follow-up	CT	−0.05	−0.12, 0.03	0.03	−1.43	0.47

MMT, Multimodal training; MET, Micro expression training; CT, Control training.

*p < 0.05, **p < 0.01, ***p < 0.001 (Holm adjusted).

For the audio modality of the ERAM, there were significant between-group differences in slopes for the pretest-posttest contrasts. The multimodal training group showed significantly larger ERA change for recognizing emotions by means of prosody (audio-only modality), both compared to the micro expression training group (between-group difference in slope = −0.11, *SE* = 0.04, *t*(109) = −2.80, *p* = 0.01; 95% CI [−0.19, −0.04]) and to the control training group (between-group difference in slope = −0.17, *SE* = 0.04, *t*(109) = −4.27, *p* < 0.001; 95% CI [−0.25, −0.09]). The effect size for the comparison with the micro expression training group was large (*d* = 1.00), and very large (*d* = 1.80) for the comparison with the control training group. There was even a significant pretest-follow-up contrast for the comparison between multimodal training group and control training group (between-group difference in slope = −0.11, *SE* = 0.04, *t*(109) = −2.54, *p* = 0.01; 95% CI [−0.19, −0.03]), but not for the comparison with the micro expression training group. The effect can be interpreted as large (*d* = 1.00).

Also in the analysis including the video-only modality of the ERAM, the between-groups differences in slopes for the pretest-posttest changes were significant and of large to very large size. The multimodal training group was superior in recognizing emotions based on only visual input, between-group difference in slope_MMT-MET_ = −0.10, *SE* = 0.04, *t* (109) = −2.55, *p* = 0.01; 95% CI [−0.18, −0.02], *d* = 0.83; between-group difference in slope_MMT-CT_ = −0.13, *SE* = 0.04, *t*(109) = −3.25, *p* < 0.001; 95% CI [−0.21, −0.05], *d* = 1.3. The same pattern as for the audio-only modality emerged when considering the pretest-follow-up change for the video-only modality. The multimodal training group was superior to the control training group (between-group difference in slope = −0.09, *SE* = 0.04, *t*(109) = −2.04, *p* = 0.04; 95% CI [−0.17, −0.00]) with a large effect size (*d* = 0.9), but not to the micro expression training group.

In the model including the combined audio-video modality, neither the pretest-posttest, nor the pretest-follow-up between-groups differences in slopes were significant. This was true for the comparisons with the micro expression training group and with the control training group (see Table 6). There were no significant within-group changes for any group for either time interval, suggesting that there were no significant improvements for audio-video ERA in either group (see Table 7). Generally, the ERA for audio-video items was also much higher than for the other two modalities (Table 2).

### 4.2. Micro expression ERA

Similarly, in the mixed multilevel analysis for micro expression ERA (MICRO), the conditional growth model with a random intercept, fixed slope and *training group* as time-invariant predictor had the best model fit (AIC = −164.02) compared to the unconditional means model with a random intercept (AIC = −113.62), the unconditional growth model with random intercept and fixed slope (AIC = −159.21), and the unconditional growth model with random intercept and random slope (AIC = −151.55). It also showed to be statistically different from the other models (*p* < 0.001) and the data was about 17 times more likely under this model than under the next best model, χ^2^(6) = 16.81, *p* = 0.01). At pretest, there were no group differences regarding micro expression ERA (Table 2).

**Figure 3:**
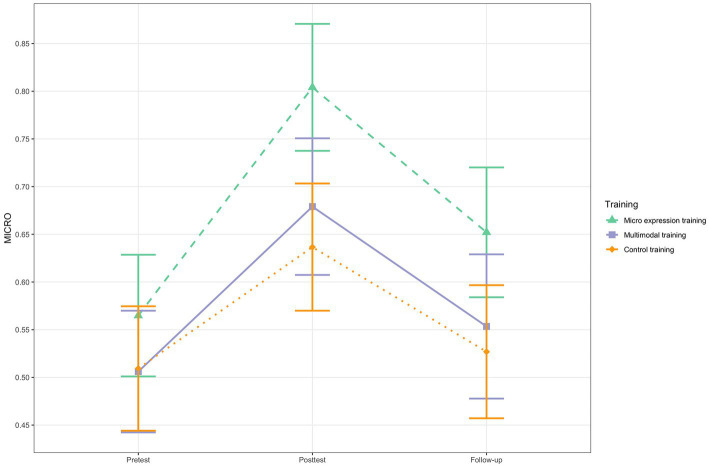
MICRO change for the three training groups. *N* = 68. Based on estimated marginal means. Error bars represent 95% Confidence Intervals.

From pretest to posttest, all three groups significantly improved in micro expression ERA. The micro expression training group increased with 24%, the multimodal training group with 17% and the control training with 13% (Table 5). There was a significant interaction effect for the pretest to follow-up contrast between the micro expression training group and the control group in so far that the micro expression training group had a significantly steeper improvement (between-group difference in slope = −0.11, *SE* = 0.06, *t*(109) = −1.96, *p* = 0.05; 95% CI [−0.22, −0.00]). The pretest-posttest difference in change scores was of moderate size (*d* = 0.69). There was no difference in micro expression ERA change trajectory between the micro expression training group and the multimodal training group (see Table 8 and Figure 3). From pretest to follow-up, only the micro expression training group’s 9% improvement was significant, even if the 95% confidence interval included zero (*M*_diff_ = −0.09, *t* (109) = −2.13, *p* = 0.04, 95% CI [−0.19, 0.01]). The other two groups did not show significant improvements from pretest to follow-up (Table 5). There were no significant differences in change trajectories from pretest to follow-up (Table 8 and Figure 3).

**Table 8:** MICRO: Fixed effects of the conditional growth model fit by maximum likelihood estimation.

	Value	*SE*	*Df*	*t*-value	*p value*	95% CI
Intercept	0.56	0.03	109	17.68	0.00***	0.50, 0.63
Time						
pre-posttest	0.24	0.04	109	5.94	0.00***	0.16, 0.32
pre-follow-up	0.09	0.04	109	2.13	0.04*	0.01, 0.17
Training						
MET vs. MMT	−0.06	0.05	65	−1.30	0.20	−0.15, 0.03
MET vs. CT	−0.06	0.05	65	−1.21	0.23	−0.14, 0.03
Interactions						
pre-posttest: MET vs. MMT	−0.07	0.06	109	−1.13	0.26	−0.18, 0.05
pre-follow-up: MET vs. MMT	−0.04	0.06	109	−0.66	0.51	−0.16, 0.08
pre-posttest: MET vs. CT	−0.11	0.06	109	−1.96	0.05*	−0.22, −0.00
pre-follow-up: MET vs. CT	−0.07	0.06	109	−1.19	0.24	−0.18, 0.04

Number of Observations: 183; Number of Groups: 68. MET, Micro expression training; MMT, Multimodal training; CT, Control training.

*p < 0.05, **p < 0.01, ***p < 0.001.

In preparatory analyses investigating possible influences on ERA (see above), we found that affective state (negative mood scale of the PANAS and positive mood scale of the IPANAT) predicted micro expression ERA at posttest. More precisely, ERA decreased the more negative/less positive the affective state was. To explore whether negative affective state influences the training groups’ ERA differently, we performed a multiple linear regression analysis for micro expression ERA at posttest using the interaction of *training group* and an aggregated score for negative state affectivity. Even if the model was significant (*adj R*^2^ = 0.29, *F* (5, 53) = 5.79, *p* < 0.001), only the main effects of negative affective state and training group predicted micro expression ERA at posttest; there were no significant interaction effects (see Supplementary Table S3), suggesting that negative affective state influenced all three groups’ multimodal ERA equally.

### 4.3. ERA for verbal and non-verbal emotional expressions In medical settings

**Figure 4:**
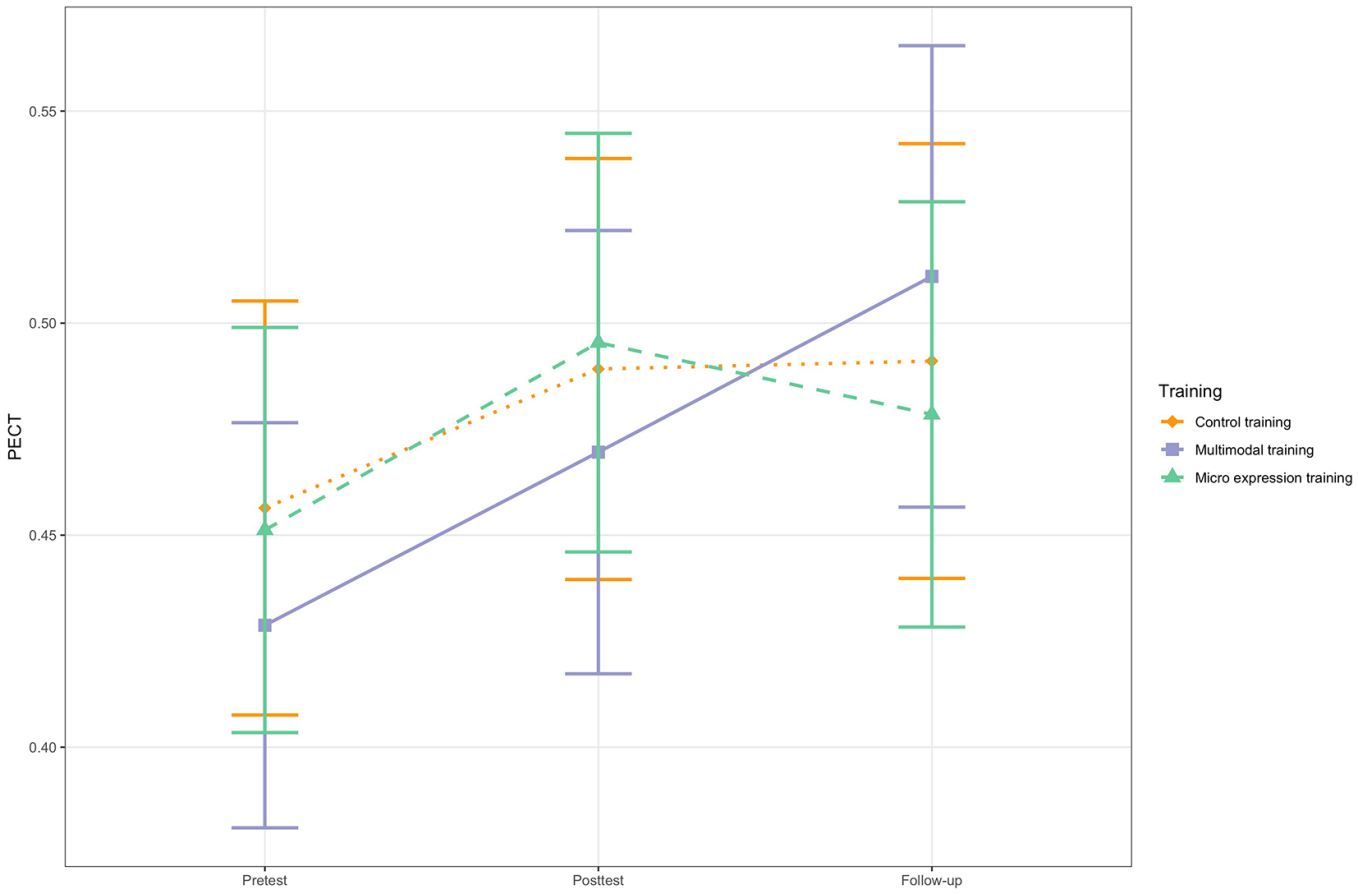
PECT change for the three training groups. *N* = 68. Based on estimated marginal means. Error bars represent 95% Confidence Intervals.

In the mixed multilevel modeling analysis for the PECT, the conditional growth model with random intercept, fixed slope of *time* and *training group* as time-invariant predictor did not show the best model fit (AIC = −306.88). Instead, the unconditional growth model with random slope and fixed intercept (AIC = −315.03) was followed by the unconditional growth model with random intercept and random slope (AIC = −313.04). Finally, the unconditional means model (AIC = −306.82) was equivalent to the conditional growth model. This suggests that training group allocation did not predict verbal and non-verbal (combined) ERA in medical contexts, even if the difference between the unconditional growth model with fixed slope and the conditional growth model was not significant (χ^2^(6) = 3.86, *p* = 0.70). Figure 4 shows the change trajectories of the groups for the PECT. Only the pretest-follow-up within-group change of the multimodal training group was significant (see Table 5) and of moderate size (*d_z_* = 0.55).

### 4.4. Baseline ERA

To investigate whether baseline ERA generally predicted ERA change scores, we conducted linear regression analyses. The results indicate that baseline ERA significantly predicts multimodal ERA change scores (*R^2^*_pretest-posttest_ = 0.07, *p* = 0.04; *R^2^*_pretest-follow-up_ = 0.10, *p* = 0.02) and micro expression ERA change scores (*R^2^*_pretest-posttest_ = 0.46, *p* < 0.001; *R^2^*_pretest-follow-up_ = 0.32, *p* < 0.001). For the PECT, only the pretest-follow-up change was significantly predicted by PECT baseline (*R^2^*_pretest-follow-up_ = 0.23, *p* < 0.001). All regressions showed the same pattern of low baseline predicting larger ERA change. The detailed results can be found in Supplementary Table S4.

To explore whether individuals who had lower baseline ERA profited from the trainings more than individuals that started out with high baseline ERA, we conducted exploratory logistic regression analyses in which we divided the participants of the multimodal training group (*n* = 18) and the micro expression training group (*n* = 21), respectively, into high and low responders. We only considered the pretest-posttest change scores for these analyses. Baseline multimodal ERA predicted the probability of whether someone would respond to the training more strongly or not, in so far that the level of baseline multimodal ERA was a significant negative predictor of the probability of being a high responder, β = −18.35, *SE* = 9.20, *p* = 0.05, 95% CI [−0.41.46, −3.68], *OR* = 0.00 [0.00, 0.03]. The participants with higher baseline multimodal ERA were the ones that responded less to the training (less pretest-posttest change) and the participants with lower baseline multimodal ERA were the ones that responded more strongly to the training (higher pretest-posttest change). For the MICRO, the trend looked similar, but the model was not significant, β = −7.25, *SE* = 4.15, *p* = 0.08, 95% CI [−0.17.54, −0.67], *OR* = 0.00 [0.00, 0.51]. The results of these analyses have to be considered with caution, as the sample sizes were very low.

## 5. Discussion

The present study is a randomized, controlled, double-blind study investigating trainee psychotherapists’ emotion recognition accuracy (ERA) one week after a three-week training period and at the one-year follow up. The multimodal ERA training led to a steeper multimodal ERA increase from pretest to posttest one week after the last training session, both compared to the micro expression ERA training and the active control training. This finding confirms that the results of a previous evaluation study including undergraduate students (Döllinger et al., 2021) could be replicated for trainee psychotherapists. When looking at the ERAM modalities separately, we can see that the effect is driven by the multimodal training group’s strong improvements in the unimodal conditions (audio-only and video-only modalities), whereas there is no between-groups difference in slopes when it comes to detecting emotions in the combined audio-video modality.

When comparing the ERA change trajectories of the groups from pretest to follow-up one year later, the picture is more complex. When considering the ERAM as total score, or when looking at the audio-video modality, the multimodal training group’s superiority does not hold up. If, however, the changes in unimodal ERA are considered, i.e., the recognition accuracies for prosody or facial expressions including body language, respectively, we see that the multimodal training group is still superior to the group that did not receive any ERA training at all (control training) and that the effect sizes were still large. But there is no effect when comparing the multimodal training group with the micro expression training group. The ERAM results suggest that a three-week (once weekly) standardized computerized training specifically targeting multimodal ERA can lead to considerable improvements in the short term (large to very large standardized effect sizes), and even in the long term, when considering the unimodal conditions. However, the within-group effects for the multimodal training group generally diminish with time and the superiority in between-group difference in slopes was only significant for the unimodal conditions, and only in comparison to the control training, not to the other ERA training. It is noteworthy that both the multimodal training group and the micro expression training group showed significant positive multimodal ERA changes from pretest to posttest and from pretest to follow-up and that the control training group did not. This suggests that the effects were not simply due to repeated testing with the ERAM, but due to something that was learned in the ERA trainings (even if the differences in slopes were not significant for the second time interval). Further, this underlines the results of a previous study (Döllinger et al., submitted) that showed that trainee psychotherapists’ ERA did not improve in response to standard PDT and CBT education (compared to a control group of undergraduate students). For the multimodal training, we also found indication to believe that baseline ERA predicts multimodal ERA and that the participants that were less good at recognizing multimodal emotional expression before the training phase profited from the training more than the ones that were better at multimodal ERA to begin with. On the one hand, this is a positive finding, as it suggests that trainee psychotherapists that possess less strong multimodal ERA competencies could improve them via explicit training. On the other hand, it cannot be ruled out that this finding could be due to a ceiling effect and should be interpreted tentatively as it is based on a very small sample size.

All three groups had considerable within-person increases in micro expression ERA from pretest to posttest and the micro expression training group even from pretest to follow-up. The mixed multilevel modeling analysis showed that micro expression ERA training led to steeper micro expression ERA change from pretest to posttest one week after the last training session, but only compared to the active control training, not compared to the multimodal training. The effect was of moderate size. The finding supports previous research showing that (trainee) psychotherapists’ micro expression ERA can be successfully trained (Johnsen, 2018; Curtis, 2021). However, there were no between-group differences in slopes from pretest to follow-up. This suggests that the standardized computerized training for micro expression ERA can lead to significant improvements in trainee psychotherapists’ micro expression ERA in the short term, at least compared to the group that did not receive any form of ERA training, but that this effect is not stable in the long term. Further, the training effect for micro expression ERA seems to be less pronounced than the training effect for multimodal ERA, based on the standardized effect sizes and between-group differences in slopes. The result is in line with previous findings of an evaluation study using an undergraduate student sample (Döllinger et al., 2021), even if the micro expression training group in the previous study displayed superiority towards both other groups. Exploratory analyses about the influence of negative affective state on micro expression ERA showed that negative affective state influenced all three groups’ micro expression ERA equally, in the sense that negative affective state led to slightly increased micro expression ERA.

The change trajectories for the third, independent outcome measure that simultaneously investigated verbal and non-verbal ERA in medical situations (PECT) were not influenced by training group allocation, neither from pretest to posttest, nor from pretest to follow-up. This shows that the multimodal and the micro expression ERA training did not lead to improvements in this additional ERA facet, i.e., there were no transfer or spill-over effects of the trainings. Apart from the long-term PECT change in the multimodal training group, there were no significant within-group contrasts at all, suggesting that ERA facets that are not explicitly trained do not improve and that ERA training needs to be targeting specific facets.

### 5.1. Implications

Previous research indicates that psychotherapists’ ERA is related to positive psychotherapy outcome and process, but that standard psychotherapy education not necessarily leads to improvements in trainee psychotherapists’ ERA. Psychotherapy programs rarely include standardized assessments for emotional competencies or other positive therapist characteristics (e.g., empathy, ability to repair alliance ruptures, self-reflective abilities) in their selection process. Thus, it might be helpful to find ways to help trainee psychotherapists to improve their ERA (among other therapist factors) during the psychotherapist education, especially those that might be struggling with emotion recognition. The present study suggests that trainee psychotherapists’ ERA can be successfully trained using standardized computerized procedures, which could be a cost-efficient and relatively timesaving way to improve ERA as part of psychotherapy education. Further, low baseline ERA was related to higher improvements, thus, trainings could, e.g., be used to support trainees that have ERA deficits. When comparing the present sample to a sample of undergraduate students that underwent the same tests and trainings (see Döllinger et al., 2021), it becomes apparent that the trainee psychotherapists, on average, had much higher ERA (see also Döllinger et al., submitted, for a review of the literature about psychotherapists’ ERA in comparison to other populations).

Whether the effects of the trainings were long-lasting is less clear. The training effects for the micro expression training, that were only moderately sized to begin with, did not hold up until follow-up one year later. This suggests that more, or another form of training for micro expression ERA might be needed to achieve in-depth changes. In the multimodal training, the superiority only persisted in the audio-only and video-only modalities, not in the audio-video modality or the total score for the ERAM. One reason for that might be that all participants displayed much larger combined audio-video ERA than for unimodal items, meaning that the task to recognize emotional expressions via both channels was too easy and that the training could not contribute to much higher ERA in that modality (even if the group differences at posttest were significant and substantial, see Table 2). On the one hand, it is positive to note that the unimodal ERA changes due to multimodal training were relatively stable. To our knowledge, this is the first study showing that standardized ERA training for psychotherapists has long-term effects. On the other hand, that the effects of the micro expression training were not long lasting, and that the longevity of effects of the multimodal training was limited to the unimodal expression/perception channels, can also be seen as a limitation of the two ERA trainings. Generally, ERA was decreasing from posttest to follow-up for the training groups. The time interval until the follow-up was very long, so we do not know when the decrease started or if it possibly could have been prevented by a booster session or another intervention. But we also have to consider the possibility that the standardized ERA trainings might not be effective enough to generate stable (trait) ERA changes, at least for micro expression ERA. Long-lasting improvements might need to be induced by more in-depth interventions or interventions that also integrate psychotherapeutic content and by that are perceived as more personally and professionally relevant by the trainees.

In general, the multimodal ERA training group showed stronger effects than the micro expression training. This might be the case because the multimodal training was experienced as more ecologically valid, as it made use of dynamic stimuli and multiple channels of expression (audio, video, audio-video). Further, according to the descriptive ERA data and ANOVAs for between-group differences at the different time points (Table 2), the largest group differences appeared in the unimodal conditions. The participants that trained in multimodal ERA were notably better at recognizing emotions that were displayed via prosody (audio only) and via facial expressions and body language (video only). Similarly, the interaction effects in the mixed multilevel models showed that only the unimodal ERAM conditions led to significantly superior improvements, especially for the pretest to follow-up comparisons. Those are conditions that might be less prevalent in everyday life, so that training those modalities might lead to particularly strong improvements. The need for explicitly training those rarer modalities might be stronger. A methodological explanation is also possible, as the micro expression outcome measure and the micro expression training made use of different item databases, whereas the multimodal training and the multimodal outcome measure used the same database (even if other items), so there could have been a habituation effect as well.

We also found indication to believe that ERA training needs to target specific ERA facets to be effective. Similar to the previous study (Döllinger et al., 2021), the ERA trainings did not lead to superior improvements in ERA in medical contexts as measured by the PECT. The multimodal training and the micro expression training were successful in improving their specific ERA facet (multimodal ERA and micro expression ERA, respectively). Still, the fact that there was no significant difference in the pretest-posttest trajectories between the micro expression training group and the multimodal training group when it comes to micro expression ERA and that the pretest-follow-up effects for unimodal ERA according to the ERAM were not differing significantly between the two training groups, leaves some room for interpretation.

We also need to discuss whether ERA as measured and trained by standardized procedures is relevant for psychotherapy. In interpersonal encounters, especially in psychotherapy, emotional expressions might not be straight forward. Patients might need help identifying and containing conflicting emotions, or simultaneous or secondary/defensive affects. The tasks and trainings used in the present study only assess single, distinct emotions and, even if the ERAM assesses more nuanced emotions, make use of stereotypical displays of emotions. Lastly, we still do not know whether standardized ERA training is impactful enough to actually influence psychotherapy results for patients. This is a question that needs to be answered empirically in future studies.

### 5.2. Strengths and limitations

The present study has many strengths, e.g., in its design. We conducted a randomized, controlled, double-blind study, that allowed us to manipulate ERA and empirically investigate the effectiveness of the ERA training interventions while controlling for test leader effects. We also tried to handle confounding variables, like gender and therapy approach, by stratified randomization, and by statistically exploring differences and influences of possible confounders, like state affect and age. The mixed design including three measurement time points for all three groups is another strength of the present study, as it allowed us to investigate ERA improvements over time and to take into account individual variability in ERA intercepts. The mixed design is also beneficial in terms of statistical power and allows us to draw more accurate conclusions. The mixed multilevel modeling approach was a suitable approach to analyze the data and handle dropouts with maximum likelihood estimation. Further, to our knowledge, it is the first ERA training study including psychotherapists or other mental health professionals that had a long-term follow-up measurement and that was using a training and a measure for multimodal ERA, and that even included the possibility to assess single modalities separately.

However, the present study also has limitations. Even if the sample size was relatively large in comparison to other studies in the field and even if the mixed design was beneficial for statistical power, we should be cautious when interpreting the group differences, as the sample sizes of the three groups were still rather small for the analyses we performed. This is particularly true for the results based on the ERAM modalities, namely the results that the unimodal auditory and unimodal visual ERA improvements were long-lasting. Also, the content of the two ERA trainings was not tailor-made for trainee psychotherapists or the psychotherapy education context, but could also be used for other populations. To reach long-lasting and practically meaningful results, training might need to be adjusted to the context. Further, the participants in the present study consisted of PDT and CBT students. This does not allow for generalizations to other therapy approaches or educations. It is very possible, that studies including trainees of other therapy approaches, e.g., approaches that explicitly focus on emotions, like *Emotion-focused Therapy* (Greenberg, 2015) or *Intensive Short Term Psychodynamic Psychotherapy* (Davanloo, 2001), might have different responses to standardized computerized ERA training. It also has to be noted that most participants seemed to know whether they belonged to one of the ERA training groups or the control group (experimental check at follow-up), leading to the conclusion that the blinding of the participants to their condition was not very successful. Thus, we cannot exclude the possibility that motivational or other factors might have influenced the participants’ ERA, e.g., that the control training group was showing less engagement and effort at the measurement occasions, since they knew that they were not receiving a real training. A methodological limitation might also be the low internal consistency of the ERAM and PECT at some time points according to Cronbach’s alpha. It is generally advisable to report several reliability estimates or composite reliability scores (Olderbak et al., 2021), however, due to a small sample, it was not possible to estimate McDonald’s omega. In a previous study (Döllinger et al., under review), the omega total values were good, suggesting a factorial structure including the twelve emotions and ERA as general factor. Laukka et al. (2021) could show good internal consistency of the ERAM according to alpha and omega. Further, it can also be questioned whether alpha is the best estimate for internal consistency in ERA tasks (e.g., due to emotion items with varying relationships, and varying intensity and difficulty levels). Nonetheless, inconsistent Cronbach’s alpha values could limit the trustworthiness and interpretability of our findings. The MICRO showed acceptable to good reliability but it has to be noted that the items in this test were less ecologically valid because the micro expressions were created using double-masked still pictures of facial expressions. The use of naturally occurring dynamic micro expressions would have been preferable. Another limitation is that some participants did not adhere to the instructions diligently, e.g., that the time intervals between the trainings were not always 1 week (even if the average was about 7 days, suggesting that most participants did follow the schedule). Lastly, the order of the ERA tasks was not counterbalanced. First, the participants conducted the MICRO, then the ERAM and then the PECT. This was also true for the ERAM modalities (first video, then audio, then audio-video combined). For that reason, we cannot preclude that learning effects or motivational effects (e.g., tiring towards the end of the session) could have influenced ERA for the different facets and modalities.

### 5.3. Future directions

To conclude, there needs to be more research about ERA training as part of psychotherapy education. The results of the present study are encouraging in that they show that it is possible to train trainee psychotherapists in multimodal and micro expression ERA using three-week, once weekly (*circa* 15 min) standardized computerized training procedures. At the same time, it is unclear how durable the effects are. The improvements due to training for the audio-only and video-only modalities of the ERAM were still detectable one year later. However, the training effects for the micro expression training and for the audio-video modality and the combined score for the ERAM were not durable. In future studies, it should be investigated at which point in time the training effects tend to diminish and if this could be avoided, e.g., by the use of a booster session or another intervention. Further, it should be investigated whether a combination of standardized ERA training for trainees with low ERA and other forms of ERA training or education for all trainees could lead to long-lasting improvements even in micro expression and multimodal audio-video ERA. Generally, it is of interest to explore which kinds of ERA training could be facilitated in psychotherapy education to produce long-lasting effects, like research about more professionally relevant interventions or other interventions that stimulate deep learning, e.g., deliberate practice and other work with video recordings of own therapy interactions and patients’ emotions that are analyzed and discussed in supervision or group seminars. However, we still think that standardized training could have a part in psychotherapy education, maybe in combination with other interventions, e.g., for trainees with lower baseline ERA in so far that they could get help in reaching a comparable ERA level to other trainees. Standardized ERA training could stimulate interest and attention towards other people’s non-verbal expressions of inner states. This idea would need to be tested empirically though.

Further, the specifics of standardized ERA trainings could be researched even further. There should be research about how to individualize training, e.g., for trainees that only have deficits in certain ERA facets, or for tailor-made trainings for specific psychotherapy settings, e.g., specifically training auditory ERA for psychotherapists working with a lying-down setting in classical psychoanalysis, or for those providing telehealth interventions. Emotion recognition accuracy training should also be applied to other psychotherapist populations, like experienced psychotherapists or psychotherapists that train in other approaches than PDT or CBT, to be able to generalize the present findings. Research should also continue to investigate factors that could be relevant for ERA and ERA trainability (e.g., affective state or emotion regulation), or training of related concepts (e.g., empathy training in psychotherapy education). In addition, more complex emotions and emotional expressions (e.g., secondary emotions, conflicting emotions) should receive attention in ERA research, especially in psychotherapy contexts.

Even if there is good indication to believe that therapists’ ERA is relevant for psychotherapy results, there needs to be more research to establish this with certainty. It is also not clear whether ERA as assessed and trained *via* standardized procedures is relevant for the work with emotion in psychotherapy. Abargil and Tishby (2021) found that the results of a standardized ERA task were related to several psychotherapy outcome and process variables, but there needs to be more research on that. In addition, it also needs to be investigated empirically whether standardized ERA training actually has an impact on psychotherapy process and outcome for patients, and, thus, is warranted at all. And finally, even if it is very relevant to assess non-verbal ERA, ultimately, there should be a simultaneous assessment of verbal and non-verbal aspects of communication in therapy. In real life therapy, patients’ verbal and non-verbal expressions of emotion are perceived simultaneously, and future research should even concentrate on the interplay of verbal and non-verbal expressions of emotion in psychotherapy.

## Data availability statement

The raw data supporting the conclusions of this article will be made available by the authors, without undue reservation.

## Ethics statement

The studies involving human participants were reviewed and approved by Stockholm Regional Ethical Review Board. The patients/participants provided their written informed consent to participate in this study.

## Author contributions

LD, LH, PL, TB, IM, HF, and SH conceptualized the idea, participated in study planning, and commented on the manuscript. LD was primarily responsible for pre-registration, data collection, data analysis, and writing of the manuscript. SH was the primary investigator. PL, LH, and TB were responsible for the programming of the trainings and tests. PL helped with data analysis. All authors contributed to the article and approved the submitted version.

## Funding

The study was financed by a research grant by the Marcus and Amalia Wallenberg Foundation (Marcus och Amalia Wallenbergs minnesfond; grant no. MAW 2013.0130). The foundation did not influence the study design, conduction or results in any way. The fees for open access publication were provided by Stockholm University.

## Conflict of interest

The authors declare that the research was conducted in the absence of any commercial or financial relationships that could be construed as a potential conflict of interest.

## Publisher’s note

All claims expressed in this article are solely those of the authors and do not necessarily represent those of their affiliated organizations, or those of the publisher, the editors and the reviewers. Any product that may be evaluated in this article, or claim that may be made by its manufacturer, is not guaranteed or endorsed by the publisher.

## Footnotes

^1^ https://osf.io/3y2gb/
