# Supplementary material for: Trainee psychotherapists’ emotion recognition accuracy improves after training: emotion recognition training as a tool for psychotherapy education
Source: Front Psychol. 2023 Jul 20;14:1188634. doi: 10.3389/fpsyg.2023.1188634 (PMC10402901; doi:10.3389/fpsyg.2023.1188634)
Supplement: Supplementary file 1 [file Data_Sheet_1.PDF]

# *Supplementary Material*

# Trainee psychotherapists' emotion recognition accuracy improves after training: Emotion recognition training as a tool for psychotherapy education

**Lillian Döllinger*, Lennart Högman, Petri Laukka, Tanja Bänziger, Irena Makower, Håkan Fischer, & Stephan Hau**

*** Correspondence:** Corresponding Author: lillian.dollinger@psychology.su.se

In the Supplementary Material, the reader finds additional analyses not reported in the manuscript. We investigated the influence of state affectivity (*Supplementary Table* 1) and the influence of age and gender on the ERA variables (*Supplementary Table 2)* using linear regression analyses. *Supplementary Table 3* presents the results of a multiple linear regression analysis to explore a possible interaction effect of training group and negative affective state on micro expression ERA at posttest. *Supplementary Table 4* shows the results of simple linear regression analyses for the influence of ERA baseline scores on the ERA change scores. *Supplementary Table 5* reports linear regression analyses exploring whether psychotherapy approach (psychodynamic psychotherapy, PDT, and cognitive behavior therapy, CBT) predicts ERA at the three time points. *Supplementary Figures 1, 2 and 3* show the individual ERA change trajectories of the participants (observed data).

## 1 The influence of state affectivity, age and gender on ERA

We performed linear regression analyses to test for possible influences of affective state (*Supplementary Table 1*), and age and gender (*Supplementary Table 2)* on ERA. We found that negative affectivity according to the PANAS influenced the ERAM scores at pretest. Since there were no ERAM group differences at pretest, we did not investigate this further. The negative affect scale of the PANAS and the positive affect scale of the IPANAT predicted micro expression ERA at posttest. See *Supplementary Table 3* for a further exploration of this. In the present study, we found that participants' age might have had an influence on the PECT scores at the pretest. However, since this was the only significant age or gender prediction, the finding should not be overstated.

**Supplementary Table 1**

*Linear regression analyses of the influence of state affectivity on the ERA variables*

| IV | DV | *b* [95% CI] | *SE b* | *β* [95% CI] | $R^2$ [95% CI] |
|---|---|---|---|---|---|
| PANAS pos pre | ERAM pretest | .02 [-.02, .06] | .02 | .12 [-.13, .37] | .01 [.00, .12] |
| PANAS pos post | ERAM posttest | -.03 [-.08, .03] | .03 | -.13 [-.40, .13] | .02 [.00, .13] |
| PANAS pos follow-up | ERAM follow-up | -.01 [-.06, .05] | .03 | -.04 [-.31, .24] | .00 [.00, .07] |
| PANAS neg pre | ERAM pretest | -.06* [-.12, -.00] | .03 | -.26 [-.51, -.01] | .07 [.00, .21] |
| PANAS neg post | ERAM posttest | -.01 [-.08, .05] | .03 | -.06 [-.32, .21] | .00 [.00, .09] |
| PANAS neg follow-up | ERAM follow-up | .04 [-.03, .11] | .03 | .16 [-.11, .43] | .03 [.00, .15] |
| PANAS pos pre | MICRO pretest | .00 [-.07, .07] | .00 | .00 [-.25, .25] | .00 [.00, 1.00] |
| PANAS pos post | MICRO posttest | .01 [-.05, .07] | .03 | .04 [-.23, .31] | .00 [.00, .07] |
| PANAS pos follow-up | MICRO follow-up | .01 [-.09, .10] | .05 | .02 [-.26, .30] | .00 [.00, .05] |
| PANAS neg pre | MICRO pretest | .04 [-.07, .14] | .05 | .09 -.16, .35] | .00 [.00, .10] |
| PANAS neg post | MICRO posttest | -.08* [.00, .16] | .04 | .27 [.01, .52] | .07* [.00, .22] |
| PANAS neg follow-up | MICRO follow-up | .08 [-.03, .19] | .06 | .19 [-.08, .46] | .04 [.00, .17] |
| PANAS pos pre | PECT pretest | -.00 [-.05, .04] | .02 | -.03 [-.28, .23] | .00 [.00, .06] |
| PANAS pos post | PECT posttest | .03 [-.03, .09] | .03 | .14 [-.13, .41] | .02 [.00, .14] |
| PANAS pos follow-up | PECT follow-up | -.00 [-.06, .05] | .03 | -.02 [-.29, .26] | .00 [.00, .05] |
| PANAS neg pre | PECT pretest | -.01 | .03 | -.03 | .00 |

| | | | | | |
|---|---|---|---|---|---|
| | | [-.08, .06] | | [-.28, .23] | [.00, .06] |
| PANAS neg post | PECT posttest | -.03 | .04 | -.11 | .01 |
| | | [-.11, .05] | | [-.37, .16] | [.00, .12] |
| PANAS neg follow-up | PECT follow-up | -.02 | .03 | -.09 | .01 |
| | | [-.09, .04] | | [-.36, .19] | [.00, .11] |
| IPANAT pos pre | ERAM pretest | -.05 | .03 | -.20 | .04 |
| | | [-.11, .01] | | [-.45, .06] | [.00, .17] |
| IPANAT pos post | ERAM posttest | -.01 | .04 | -.04 | .00 |
| | | [-.10, .07] | | [-.31, .23] | [.00, .08] |
| IPANAT pos follow-up | ERAM follow-up | -.00 | .05 | -.01 | .00 |
| | | [-.11, .10] | | [-.30, .28] | [.00, .03] |
| IPANAT neg pre | ERAM pretest | -.00 | .03 | -.02 | .00 |
| | | [-.06, -.05] | | [-.29, .24] | [.00, .05] |
| IPANAT neg post | ERAM posttest | .04 | .05 | .12 | .02 |
| | | [-.05, .13] | | [-.16, .41] | [.00, .13] |
| IPANAT neg follow-up | ERAM follow-up | .05 | .04 | .18 | .03 |
| | | [-.03, .14] | | [-.10, .46] | [.00, .17] |
| IPANAT pos pre | MICRO pretest | .00 | .06 | .01 | .00 |
| | | [-.11, .11] | | [-.26, .27] | [.00, .01] |
| IPANAT pos post | MICRO posttest | -.13* | .05 | -.33 | .11* |
| | | [-.24, -.03] | | [-.58, -.07] | [.00, .27] |
| IPANAT pos follow-up | MICRO follow-up | .03 | .08 | .06 | .00 |
| | | [-.14, .20] | | [-.23, .34] | [.00, .09] |
| IPANAT neg pre | MICRO pretest | .02 | .05 | .05 | .00 |
| | | [-.09, .13] | | -.22, .31] | [.00, .08] |
| IPANAT neg post | MICRO posttest | .10 | .06 | .23 | .05 |
| | | [-.02, .21] | | [-.05, .51] | [.00, .20] |
| IPANAT neg follow-up | MICRO follow-up | .07 | .07 | .14 | .02 |
| | | [-.07, .21] | | [-.14, .42] | [.00, .15] |
| IPANAT pos pre | PECT pretest | -.01 | .04 | -.03 | .00 |
| | | [-.08, .06] | | [-.30, .23] | [.00, .07] |
| IPANAT pos post | PECT posttest | .05 | .05 | .15 | .02 |
| | | [-.04, .15] | | [-.12, .42] | [.00, .15] |
| IPANAT pos follow-up | PECT follow-up | .07 | .05 | .21 | .04 |
| | | [-.02, .17] | | [-.07, .49] | [.00, .19] |
| IPANAT neg pre | PECT pretest | .02 | .03 | .09 | .01 |

| | | | | | |
|---|---|---|---|---|---|
| | | [-.05, .09] | | [-.18, .35] | [.00, .10] |
| IPANAT neg post | PECT posttest | .07<br>[-.04, .18] | .05 | .18<br>[-.10, .46] | .03<br>[.00, .17] |
| IPANAT neg follow-up | PECT follow-up | .04<br>[-.05, .12] | .04 | .13<br>[-.15, .42] | .02<br>[.00, .14] |

*Note. b* represents unstandardized regression weights; b*eta* indicates the standardized regression weights. **p* < .05, ***p* < .01

**Supplementary Table 2**

*Simple linear regression analyses of the influence of age and gender on the ERA variables*

| IV | *DV* | *b*<br>[95% CI] | *SE b* | $\beta$<br>[95% CI] | $R^2$<br>[95% CI] |
|---|---|---|---|---|---|
| Age | ERAM pretest | .00<br>[-.00, .00] | .00 | .05<br>[-.19, .30] | .00<br>[.00, .07] |
| Age | ERAM posttest | .00<br>[-.00, .01] | .00 | .16<br>[-.10, .42] | .03<br>[.00, .14] |
| Age | ERAM follow-up | -.00<br>[-.01, .00] | .00 | -.03<br>[-.30, .25] | .00<br>[.00, .06] |
| Age | MICRO pretest | -.00<br>[-.01, .01] | .00 | -.00<br>[-.25, .24] | .00<br>[.00, .01] |
| Age | MICRO posttest | -.00<br>[-.01, .00] | .00 | -.06<br>[-.33, .20] | .00<br>[.00, .09] |
| Age | MICRO follow-up | -.01<br>[-.01, .00] | .00 | .18<br>[-.45, .09] | .03<br>[.00, .17] |
| Age | PECT pretest | -.00*<br>[-.01, -.00] | .00 | -.28<br>[-.52, -.04] | .08*<br>[.00, .22] |
| Age | PECT posttest | -.00<br>[-.01, .00] | .00 | -.21<br>[-.47, .05] | .05<br>[.00, .18] |
| Age | PECT follow-up | -.00<br>[-.01, .00] | .00 | -.19<br>[-.47, .08] | .04<br>[.00, .18] |
| Gender | ERAM pretest | .01<br>[-.03, .05] | .02 | .05<br>[-.19, .30] | .00<br>[.00, .07] |
| Gender | ERAM posttest | .01<br>[-.05, .07] | .03 | .05<br>[-.21, .31] | .00<br>[.00, .08] |

| | | | | | |
|---|---|---|---|---|---|
| Gender | ERAM follow-up | -.01 [-.06, .05] | .03 | -.04 [-.31, .24] | .00 [.00, .08] |
| Gender | MICRO pretest | -.03 [-.11, .05] | .04 | -.10 [-.34, .14] | .01 [.00, .10] |
| Gender | MICRO posttest | -.01 [-.08, .07] | .04 | -.02 [-.29, .24] | .00 [.00, .06] |
| Gender | MICRO follow-up | -.04 [-.14, .05] | .05 | -.12 [-.40, .15] | .02 [.00, .13] |
| Gender | PECT pretest | .01 [-.04, .06] | .03 | .04 [-.21, .29] | .00 [.00, .07] |
| Gender | PECT posttest | .04 [-.03, .11] | .04 | .14 [-.13, .40] | .02 [.00, .13] |
| Gender | PECT follow-up | -.01 [-.06, .05] | .03 | -.03 [-.31, .24] | .00 [.00, .07] |

*Note. b* represents unstandardized regression weights; b*eta* indicates the standardized regression weights. **p* < .05, ***p* < .01

## 2 Negative affective state by training group interaction effect on MICRO at posttest

Since there was indication to believe that negative state affectivity influenced the MICRO posttest scores (*Supplementary Table 1*), we explored whether training group and negative affective state together predicted the MICRO score at posttest. For that, we used an aggregated score of the PANAS negative affectivity and IPANAT positive affectivity (reversed) as a composite measure for *negative state affectivity* (based on averaged z-scores). *Supplementary Table 3* shows the results of this multiple linear regression analysis. The model was significant (*adj* $R^2$ = .29, $F(5, 53) = 5.79$, $p$ = .00), but there were no interactions between training group and negative affective state.

**Supplementary Table 3**

*Multiple linear regression analysis of the influence of training group by negative affective state on micro expression ERA at posttest*

| Predictor | *b* [95% CI] | $sr^2$ [95% CI] | *Fit* |
|---|---|---|---|
| Intercept | 0.78** | | |
| | [0.73, 0.84] | | |
| Training | | | |
| MET vs. MMT | -0.10* | .08 | |
| | [-0.18, -0.02] | [-.03, .19] | |
| MET vs. CT | -0.14** | .17 | |
| | [-0.22, -0.06] | [.01, .32] | |
| Negative affect | .08* | .06 | |
| | [0.01, 0.15] | [-.04, .15] | |
| Interactions | | | |
| MET vs. MMT: negative affect | 0.02 | .00 | |
| | [-0.10, 0.15] | [-.01, .02] | |
| MET vs. CT: negative affect | -0.04 | .01 | |
| | [-0.14, 0.07] | [-.03, .04] | |
| | | | $R^2$ = .35** |
| | | | [.10, .47] |
| | | | *adj.* $R^2$ = .29** |

*Note*. *N* = 59. MET = Micro expression training, MMT = Multimodal training, CT = Control training. A significant *b*-weight indicates the semi-partial correlation is also significant. *b* represents unstandardized regression weights. $sr^2$ represents the semi-partial correlation squared. Table created with *APAtables*. (*R* Studio). **p* < .05, ***p* < .01

## 3 Influence of baseline ERA on ERA change values

*Supplementary Table 4* shows the results of simple linear regression analyses about the influence of ERA baseline on ERA change scores. The centered baseline scores predict both ERA change scores for the ERAM and the MICRO, and the pretest–follow-up change score for the PECT.

**Supplementary Table 4**

*Simple linear regression analyses of the influence of ERA baseline values on ERA change*

| IV | *DV* | *b* [95% CI] | *SE b* | *β* [95% CI] | $R^2$ [95% CI] |
|---|---|---|---|---|---|
| ERAM baseline | ERAM change pretest–posttest | -.28* [-.55, -.01] | .14 | -.26 [-.52, -.01] | .07* [.00, .22] |
| ERAM baseline | ERAM change pretest–follow-up | -.29* [-.53, -.05] | .12 | -.32 [-.58, -.05] | .10* [.00, .26] |
| MICRO baseline | MICRO change pretest–posttest | -.76** [-.97, -.54] | .11 | -.67 [-.87, -.48] | .46** [.26, .59] |
| MICRO baseline | MICRO change pretest–follow-up | -.63** [-.89, -.38] | .13 | -.56 [-.79, -.33] | .32** [.12, .48] |
| PECT baseline | PECT change pretest–posttest | -.13 [-.39, .12] | .14 | -.14 [-.41, -.12] | .02 [.00,.14] |
| PECT baseline | PECT change pretest–follow-up | -.42** [-.62, -.21] | .10 | -.48 [-.73, -.24] | .23** [.06,.41] |

*Note. b* represents unstandardized regression weights; b*eta* indicates the standardized regression weights.

*$p < .05$, **$p < .01$

## 4 Differences between PDT and CBT students

In the present study, we did not differentiate between PDT and CBT students. We consider ERA to be a common factor in psychotherapy, i.e., that it is relevant for psychotherapists of all approaches to be good at detecting patients' non-verbal emotional expression. However, for the interested readers, we explored the possible influence of psychotherapy approach on ERA. There was no significant influence of psychotherapy approach on the ERAM or MICRO, but on the PECT score at follow-up (*Supplementary Table 5*). The CBT students had significantly higher verbal and non-verbal (combined) ERA in medical settings (according to the PECT) at follow-up (CBT: $M$ = .54, $SD$ = .10, 95% CI [.49, .58], $n$ = 22; PDT: $M$ = .47, $SD$ = .09, 95% CI [.44, .50], $n$ = 39; $t(53) = -2.57$, $p$ = .01, 95% CI [-.12, -.01]) according to two-sided *t*-tests. This could indicate that the CBT psychotherapy education contributes to increased ERA for medical situations when both verbal and nonverbal content is displayed. Since the PECT was not a main focus of the present study (none of the training groups trained to recognize emotional expressions in medical settings) and as to not lose statistical power, we did not conduct any additional analyses differentiating between CBT and PDT students.

**Supplementary Table 5**

*Simple linear regression analyses of the influence of psychotherapy approach (PDT or CBT) on the ERA variables*

| IV | *DV* | *b* [95% CI] | *SE b* | *β* [95% CI] | $R^2$ [95% CI] |
|---|---|---|---|---|---|
| Psychotherapy approach | ERAM pretest | -.00 [-.05, .04] | .02 | -.01 [-.26, .23] | .00 [.00, .04] |
| Psychotherapy approach | ERAM posttest | .01 [-.05, .07] | .03 | .05 [-.21, .31] | .00 [.00, .08] |
| Psychotherapy approach | ERAM follow-up | .01 [-.05, .07] | .03 | .05 [-.22, .33] | .00 [.00, .09] |
| Psychotherapy approach | MICRO pretest | -.02 [-.10, .06] | .04 | -.07 [-.31, .18] | .01 [.00, .08] |
| Psychotherapy approach | MICRO posttest | .02 [-.09, .06] | .04 | -.06 [-.32, .20] | .00 [.00,.09] |
| Psychotherapy approach | MICRO follow-up | -.01 [-.11, .09] | .05 | -.03 [-.30, .25] | .00 [.00,.06] |
| Psychotherapy approach | PECT pretest | .05 [-.00, .10] | .02 | .24 [-.00, .48] | .06 [.00,.19] |
| Psychotherapy approach | PECT posttest | .06 [-.01, .13] | .03 | .21 [-.05, .47] | .04 [.00,.18] |
| Psychotherapy approach | PECT follow-up | .07* [.01, .12] | .03 | .33 [.07, .59] | .11 [.00,.28] |

*Note. b* represents unstandardized regression weights; b*eta* indicates the standardized regression weights.

**p* < .05, ***p* < .01

## 5 Individual ERA trajectories

In *Supplementary Figures 1, 2* and 3, the reader finds the ERA trajectories of the participants (observed data). The trajectories of the participants were very similar; most improved from pretest to posttest and then deteriorated again until follow-up.

**Supplementary figure 1**

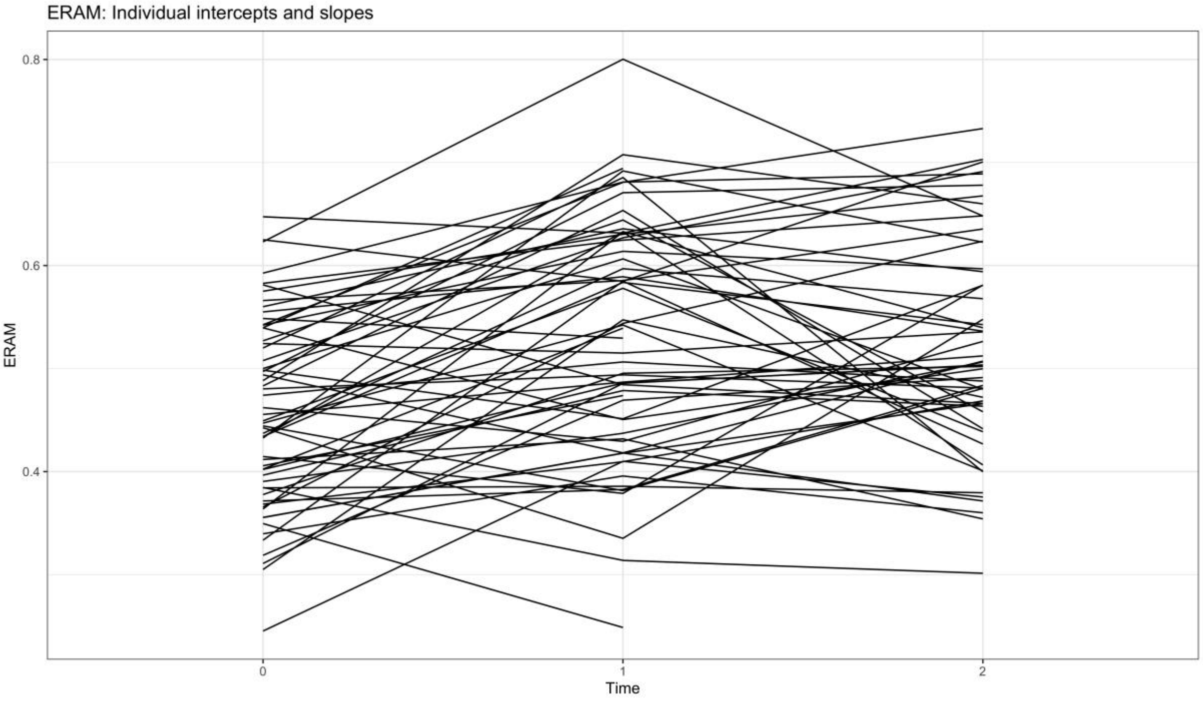


Note. N = 68. Time: 0 = pretest, 1 = posttest, 2 = follow-up.

**Supplementary figure 2**

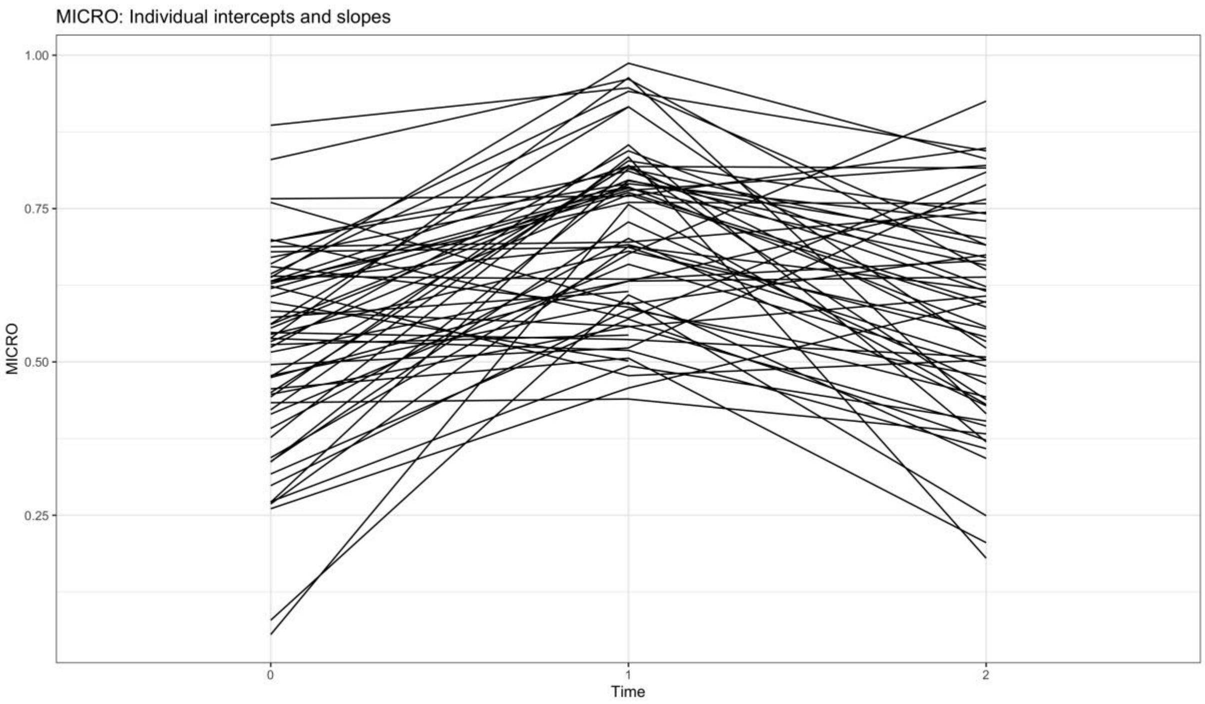


Note. N = 68. Time: 0 = pretest, 1 = posttest, 2 = follow-up.

**Supplementary figure 3**

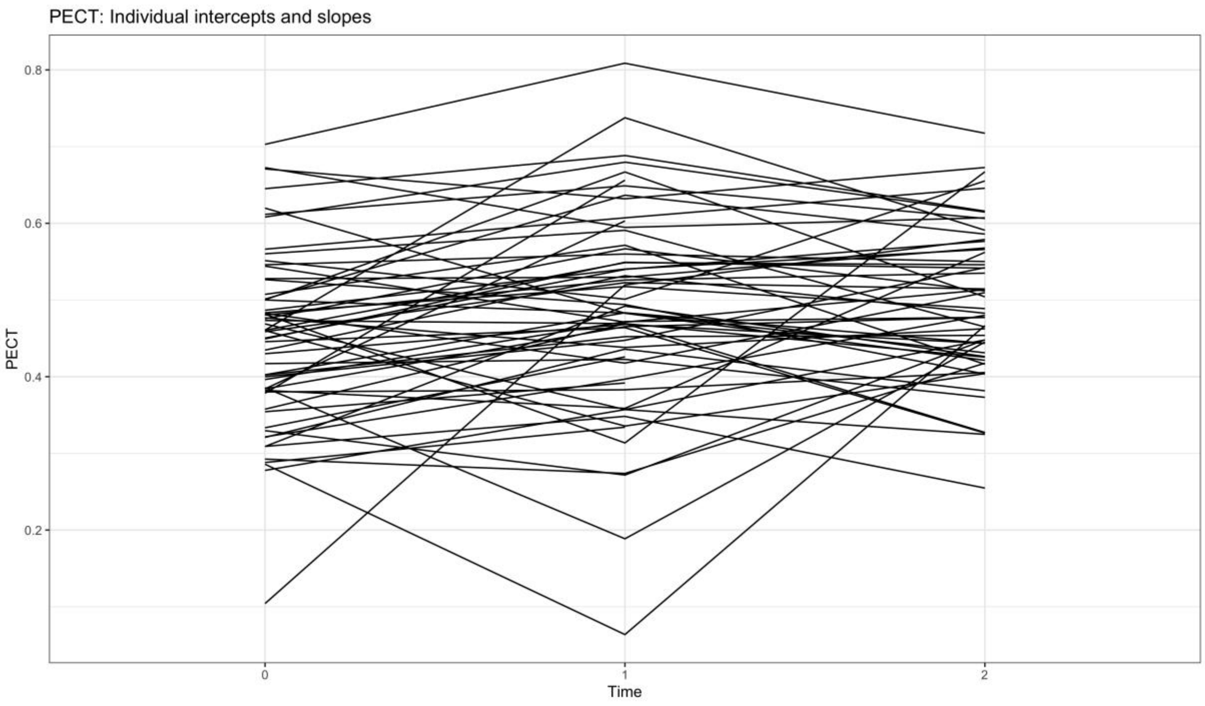


Note. N = 68. Time: 0 = pretest, 1 = posttest, 2 = follow-up.
